# Supplementary material for: Geopolitical risk contagion across strategic sectors: Nonlinear evidence from defense, cybersecurity, energy, and raw materials
Source: PLoS One. 2025 Sep 2;20(9):e0330557. doi: 10.1371/journal.pone.0330557 (PMC12404389; doi:10.1371/journal.pone.0330557)
Supplement: S1 Table — (DOCX) [file pone.0330557.s001.docx]

**Table 1. Sectoral behavior based on the distribution of returns conditioned by GPR**

| **Sector** | **Quantile 5%** | **Quantile 50%** | **Quantile 95%** | **Sectoral sensitivity pattern to GPR** |
| --- | --- | --- | --- | --- |
| **Defense** | High | Moderate | High | High sensitivity across regimes; pro-cyclical returns |
| **Cybersecurity** | Moderate | High | Very high | Highly asymmetric; speculative growth under stress |
| **Energy** | High | Moderate | Low/Moderate | Bidirectional exposure; regime-dependent |
| **Raw materials** | Moderate | Moderate/Stable | Moderate | Conditional on supply chain and geopolitical trade tensions |
| **ETFs** | Moderate | Moderate/Stable | Moderate | Balanced performance; low-to-moderate sensitivity to GPR |
| Note: The values presented reflect the average returns of representative companies within each sector, estimated using the QQR method across the 5th, 50th, and 95th quantiles of the conditional distribution based on the GPR component. Qualitative descriptors were assigned using the following thresholds: “Very high” > 0.25; “High” = 0.15–0.25; “Moderate” = 0.05–0.15; “Low” ≤ 0.05. The "Sensitivity to GPR" column summarizes the dominant response pattern across regimes. See Appendices S5 and S6 for full heatmaps. | | | | |
