# Supplementary material for: Geopolitical risk contagion across strategic sectors: Nonlinear evidence from defense, cybersecurity, energy, and raw materials
Source: PLoS One. 2025 Sep 2;20(9):e0330557. doi: 10.1371/journal.pone.0330557 (PMC12404389; doi:10.1371/journal.pone.0330557)
Supplement: S2 Table — (DOCX) [file pone.0330557.s002.docx]

**Table 2. Volatility profile of strategic sectors according to the quantiles of the conditional distribution**

| **Sector** | **Quantile 5%** | **Quantile 50%** | **Quantile 95%** | **Risk profile** |
| --- | --- | --- | --- | --- |
| **Defense** | Low/Moderate | Moderate | High | Stable under normal conditions, reactive during crises |
| **Cybersecurity** | Moderate/High | Very high | Extreme | Unstable and volatile across all regimes, speculative under stress |
| **Energy** | Variable/High | High/Unstable | Very high | Highly exposed to systemic stress |
| **Raw materials** | Moderate | Unstable | Very high | Elevated and unstable, especially under geopolitical stress |
| **ETFs** | Low | Low/Moderate | Moderate | Defensive, with low risk across all regimes |
| Note: The values presented reflect the relative levels of sectoral volatility, estimated using the QQR method across the 5th, 50th, and 95th quantiles of the conditional distribution based on the GPR component. Qualitative descriptors were assigned using the following thresholds: “Extreme” > 0.30; “Very high” = 0.20–0.30; “Moderate/high” = 0.10–0.20; “Low” ≤ 0.10. The "Risk Profile" column summarizes each sector’s exposure to systemic stress and sensitivity to tail-risk regimes. See Appendices S5 and S6 for full heatmaps. | | | | |
