# Supplementary material for: Geopolitical risk contagion across strategic sectors: Nonlinear evidence from defense, cybersecurity, energy, and raw materials
Source: PLoS One. 2025 Sep 2;20(9):e0330557. doi: 10.1371/journal.pone.0330557 (PMC12404389; doi:10.1371/journal.pone.0330557)
Supplement: S1 Appendix — (DOCX) [file pone.0330557.s003.docx]

| **S1 Appendix. List of analyzed companies and corresponding symbols.** | | |
| --- | --- | --- |
| **Industry focus** | **Company** | **Symbol** |
| Defense & Aerospace | Lockheed Martin Corporation | LMT |
|  | Rtx Corp | RTX |
|  | Northrop Grumman Corporation | NOC |
|  | Boeing Co | BA |
|  | General Dynamics Corporation | GD |
|  | BAE Systems PLC | BAES |
|  | AviChina Industry & Technology Co Ltd | V2357 |
|  | L3Harris Technologies Inc | LHX |
|  | Airbus Group SE | AIR |
|  | Leonardo SpA | LDOF |
|  | Thales | TCFP |
|  | Huntington Ingalls Industries Inc | HII |
|  | Leidos Holdings Inc | LDOS |
|  | Booz Allen Hamilton Holding | BAH |
|  | Rolls-Royce Holdings PLC | RR |
|  | CACI International Inc | CACI |
|  | Rheinmetall AG | RHMG |
|  | Elbit Systems Ltd | ESLT |
|  | Honeywell International Inc | HON |
|  | GE Aerospace | GE |
|  | Safran SA | SAF |
|  | SAAB AB ser. B | SAABBs |
|  | KBR Inc | KBR |
|  | Babcock International Group PLC | BAB |
|  | Mitsubishi Heavy Industries, Ltd. | V7011 |
|  | Science Applications International Corp | SAIC |
|  | Dassault Aviation SA | AM |
|  | Textron Inc | TXT |
|  | Parker-Hannifin Corporation | PH |
|  | Transdigm Group Incorporated | TDG |
|  | Aselsan Elektronik Sanayi ve Ticaret AS | ASELS |
|  | Jacobs Engineering Group Inc | J |
|  | Korea Aerospac | V047810 |
|  | Serco Group | SRP |
|  | Singapore Tech Engineering Ltd | STEG |
|  | Teledyne Technologies Incorporated | TDY |
|  | Oshkosh Corporation | OSK |
|  | Kawasaki Heavy Industries, Ltd. | V7012 |
|  | Thyssenkrupp AG O.N. | TKAG |
|  | Bharat Electronics Ltd | BAJE |
| Cybersecurity | Microsoft Corporation | MSFT |
|  | Broadcom Inc | AVGO |
|  | Cisco Systems Inc | CSCO |
|  | International Business Machines | IBM |
|  | Palo Alto Networks Inc | PANW |
|  | Fortinet Inc | FTNT |
|  | Check Point Software Technologies Ltd | CHKP |
|  | Accenture plc | ACN |
|  | Akamai Technologies Inc | AKAM |
|  | F5 Networks Inc | FFIV |
|  | NetScout Systems Inc | NTCT |
|  | Radware Ltd | RDWR |
|  | Trend Micro Inc. | 4704 |
|  | BlackBerry Ltd | BB |
|  | Juniper Networks Inc | JNPR |
|  | Qualys Inc | QLYS |
|  | Trend Micro Inc ADR | TMICY |
|  | Allot Ltd | ALLT |
|  | Parrot | PARRO |
| Energy & Strategic Resources | Exxon Mobil Corp | XOM |
|  | LUKOIL PJSC | LKOH |
|  | Phillips 66 | PSX |
|  | China Shenhua Energy Co Ltd | CSUAY |
|  | Valero Energy Corporation | VLO |
|  | Reliance Industries Ltd | RELI |
|  | TotalEnergies SE ADR | TTE |
|  | China Petroleum & Chemical Corp Class H | V0386 |
|  | PTT PCL | PTT |
|  | Marathon Petroleum Corp | MPC |
|  | Indian Oil Corporation Ltd | IOC |
|  | Chevron Corp | CVX |
|  | Equinor ASA ADR | EQNR |
|  | Shell PLC ADR | SHEL |
|  | Gazprom PJSC | GAZP |
|  | Nextera Energy Inc | NEE |
|  | National Grid PLC ADR | NGG |
|  | Enbw Energie Baden Wuerttemberg AG | EBKG |
|  | Oil And Natural Gas Corporation Ltd | ONGC |
|  | Suncor Energy Inc | SU |
|  | Exelon Corporation | EXC |
|  | RWE AG ST O.N. | RWEG |
|  | Eni SpA ADR | E |
|  | CNOOC Ltd | V0883 |
| Defense-focused ETFs and Investment Funds | iShares U.S. Aerospace & Defense ETF | ITA |
|  | SPDR® S&P Aerospace & Defense ETF | XAR |
|  | Invesco Aerospace & Defense ETF | PPA |
|  | Vanguard Industrials Index Fund ETF Shares | VIS |
|  | iShares U.S. Industrials ETF | IYJ |
|  | Fidelity MSCI Industrials Index ETF | FIDU |
|  | iShares Global Industrials ETF | EXI |
| Note: S1 Appendix lists the companies and corresponding symbols analyzed, categorized by industry: defense & aerospace, cybersecurity, energy & strategic resources, and defense-focused ETFs. | | |
