# Supplementary material for: Geopolitical risk contagion across strategic sectors: Nonlinear evidence from defense, cybersecurity, energy, and raw materials
Source: PLoS One. 2025 Sep 2;20(9):e0330557. doi: 10.1371/journal.pone.0330557 (PMC12404389; doi:10.1371/journal.pone.0330557)
Supplement: S2 Appendix — (DOCX) [file pone.0330557.s004.docx]

| **S2 APPENDIX Descriptive statistics** | | | | | | | | | |
| --- | --- | --- | --- | --- | --- | --- | --- | --- | --- |
|  | **Mean** | **Median** | **Max.** | **Min.** | **Std. Dev.** | **Skew.** | **Kurt** | **JB** | **Prob.** |
| **LMT** | 0.036 | 0.064 | 10.191 | $-$13.653 | 1.394 | $-$0.705 | 18.34 | 27 382.55 | 0.000 |
| **RTX** | 0.022 | 0.030 | 14.620 | $-$15.644 | 1.635 | $-$0.339 | 18.62 | 28 202.15 | 0.000 |
| **NOC** | 0.048 | 0.081 | 12.223 | $-$10.699 | 1.500 | $-$0.042 | 11.18 | 7 728.90 | 0.000 |
| **BA** | 0.012 | 0.034 | 21.768 | $-$27.244 | 2.454 | $-$0.516 | 21.58 | 39 951.19 | 0.000 |
| **GD** | 0.031 | 0.087 | 8.753 | $-$11.571 | 1.377 | $-$0.429 | 9.96 | 5 672.86 | 0.000 |
| **BAES** | 0.040 | 0.070 | 10.008 | $-$8.701 | 1.460 | $-$0.127 | 8.17 | 3 089.97 | 0.000 |
| **V2357** | $-$0.005 | 0.000 | 28.546 | $-$26.113 | 2.784 | 0.616 | 16.90 | 22 478.57 | 0.000 |
| **LHX** | 0.037 | 0.042 | 11.215 | $-$15.431 | 1.562 | $-$0.175 | 14.22 | 14 538.97 | 0.000 |
| **AIR** | 0.041 | 0.049 | 18.625 | $-$25.045 | 2.186 | $-$0.377 | 19.73 | 32 376.41 | 0.000 |
| **LDOF** | 0.058 | 0.071 | 15.151 | $-$25.093 | 2.320 | $-$0.755 | 16.41 | 21 016.29 | 0.000 |
| **TCFP** | 0.050 | 0.021 | 12.854 | $-$11.947 | 1.631 | 0.170 | 10.47 | 6 458.56 | 0.000 |
| **HII** | 0.020 | 0.086 | 10.809 | $-$30.328 | 1.847 | $-$2.432 | 38.87 | 151 269.50 | 0.000 |
| **LDOS** | 0.048 | 0.108 | 10.231 | $-$20.358 | 1.767 | $-$1.478 | 21.53 | 40 632.24 | 0.000 |
| **BAH** | 0.065 | 0.106 | 12.732 | $-$20.938 | 1.674 | $-$1.171 | 19.98 | 33 910.77 | 0.000 |
| **RR** | $-$0.017 | 0.000 | 36.298 | $-$108.973 | 3.548 | $-$9.920 | 330.76 | 12 444 078.00 | 0.000 |
| **CACI** | 0.054 | 0.077 | 14.011 | $-$14.921 | 1.689 | $-$0.302 | 13.91 | 13 768.23 | 0.000 |
| **RHMG** | 0.104 | 0.072 | 22.155 | $-$12.545 | 2.233 | 0.498 | 11.81 | 9 070.73 | 0.000 |
| **ESLT** | 0.059 | 0.049 | 14.431 | $-$11.162 | 1.626 | $-$0.052 | 9.04 | 4 218.45 | 0.000 |
| **HON** | 0.029 | 0.071 | 14.036 | $-$12.882 | 1.406 | $-$0.244 | 14.96 | 16 548.15 | 0.000 |
| **GE** | 0.010 | 0.000 | 13.746 | $-$19.790 | 2.160 | $-$0.389 | 11.89 | 9 183.29 | 0.000 |
| **SAF** | 0.055 | 0.044 | 19.006 | $-$25.973 | 2.032 | $-$0.636 | 27.91 | 71 815.92 | 0.000 |
| **SAABBS** | 0.075 | 0.000 | 15.440 | $-$14.536 | 1.966 | 0.033 | 11.98 | 9 301.59 | 0.000 |
| **KBR** | 0.017 | 0.059 | 16.821 | $-$26.000 | 2.349 | $-$1.183 | 20.02 | 34 086.26 | 0.000 |
| **BAB** | $-$0.024 | 0.000 | 27.751 | $-$17.906 | 2.135 | 0.396 | 19.60 | 31 869.45 | 0.000 |
| **V7011** | 0.044 | 0.000 | 18.099 | $-$16.206 | 1.998 | 0.262 | 9.77 | 5 317.37 | 0.000 |
| **SAIC** | 0.036 | 0.120 | 17.177 | $-$20.109 | 1.959 | $-$1.548 | 22.54 | 45 173.49 | 0.000 |
| **AM** | 0.033 | 0.000 | 11.521 | $-$12.134 | 1.810 | $-$0.032 | 8.23 | 3 156.36 | 0.000 |
| **TXT** | 0.024 | 0.044 | 15.015 | $-$18.789 | 2.035 | $-$0.470 | 14.67 | 15 821.09 | 0.000 |
| **PH** | 0.062 | 0.057 | 16.906 | $-$18.258 | 1.897 | $-$0.375 | 15.27 | 17 443.17 | 0.000 |
| **TDG** | 0.088 | 0.147 | 21.836 | $-$24.813 | 2.048 | $-$0.648 | 26.33 | 63 036.30 | 0.000 |
| **ASELS** | 0.163 | 0.000 | 12.235 | $-$21.519 | 2.431 | $-$0.127 | 8.34 | 3 300.40 | 0.000 |
| **J** | 0.027 | 0.044 | 9.789 | $-$17.889 | 1.723 | $-$0.670 | 12.68 | 11 017.87 | 0.000 |
| **V047810** | 0.023 | 0.000 | 16.399 | $-$35.382 | 2.508 | $-$0.975 | 21.76 | 41 065.35 | 0.000 |
| **SRP** | $-$0.026 | 0.000 | 15.319 | $-$38.857 | 2.177 | $-$2.274 | 50.11 | 258 542.50 | 0.000 |
| **STEG** | 0.011 | 0.000 | 8.408 | $-$10.447 | 1.199 | $-$0.243 | 10.39 | 6 336.62 | 0.000 |
| **TDY** | 0.060 | 0.115 | 12.504 | $-$25.990 | 1.687 | $-$1.565 | 29.62 | 82 925.22 | 0.000 |
| **OSK** | 0.022 | 0.042 | 19.401 | $-$14.871 | 2.147 | 0.182 | 10.59 | 6 659.98 | 0.000 |
| **V7012** | 0.105 | 0.000 | 226.711 | $-$15.998 | 4.894 | 35.854 | 1 661.02 | 318 000 000.00 | 0.000 |
| **TKAG** | $-$0.042 | 0.000 | 24.846 | $-$9.991 | 2.877 | 0.184 | 13.01 | 11 586.58 | 0.000 |
| **BAJE** | 0.119 | 0.000 | 16.451 | $-$22.068 | 2.357 | $-$0.138 | 11.02 | 7 436.72 | 0.000 |
| **MSFT** | 0.086 | 0.083 | 13.293 | $-$15.945 | 1.672 | $-$0.176 | 11.17 | 7 708.97 | 0.000 |
| **AVGO** | 0.129 | 0.124 | 21.859 | $-$22.186 | 2.354 | $-$0.202 | 14.26 | 14 642.46 | 0.000 |
| **CSCO** | 0.038 | 0.053 | 12.552 | $-$14.769 | 1.545 | $-$0.633 | 15.22 | 17 426.18 | 0.000 |
| **IBM** | 0.013 | 0.052 | 12.190 | $-$13.755 | 1.503 | $-$0.491 | 14.30 | 14 849.68 | 0.000 |
| **PANW** | 0.100 | 0.169 | 17.060 | $-$33.458 | 2.487 | $-$1.510 | 25.58 | 59 880.26 | 0.000 |
| **FTNT** | 0.116 | 0.189 | 22.554 | $-$28.856 | 2.470 | $-$0.869 | 20.64 | 36 275.92 | 0.000 |
| **CHKP** | 0.042 | 0.061 | 13.245 | $-$15.629 | 1.479 | $-$0.755 | 17.02 | 22 955.33 | 0.000 |
| **ACN** | 0.053 | 0.110 | 12.095 | $-$9.770 | 1.528 | $-$0.052 | 9.71 | 5 200.45 | 0.000 |
| **AKAM** | 0.009 | 0.113 | 19.230 | $-$24.498 | 1.989 | $-$1.359 | 27.47 | 69 976.19 | 0.000 |
| **FFIV** | 0.036 | 0.083 | 12.213 | $-$13.744 | 1.811 | $-$0.341 | 10.31 | 6 221.46 | 0.000 |
| **NTCT** | $-$0.016 | 0.000 | 13.045 | $-$23.708 | 2.175 | $-$1.014 | 17.12 | 23 500.90 | 0.000 |
| **RDWR** | 0.012 | 0.040 | 20.283 | $-$21.209 | 1.985 | $-$0.812 | 20.41 | 35 293.23 | 0.000 |
| **V4704** | 0.044 | 0.000 | 14.885 | $-$18.874 | 1.896 | 0.115 | 15.20 | 17 185.34 | 0.000 |
| **BB** | $-$0.019 | $-$0.101 | 28.265 | $-$53.843 | 3.658 | $-$0.684 | 29.75 | 82 783.15 | 0.000 |
| **JNPR** | 0.010 | 0.065 | 19.727 | $-$16.692 | 1.724 | $-$0.185 | 16.71 | 21 694.21 | 0.000 |
| **QLYS** | 0.059 | 0.164 | 21.652 | $-$39.706 | 2.696 | $-$1.546 | 28.30 | 74 984.00 | 0.000 |
| **TMICY** | 0.030 | 0.000 | 15.956 | $-$17.249 | 1.871 | 0.272 | 11.34 | 8 071.03 | 0.000 |
| **ALLT** | $-$0.032 | 0.000 | 23.726 | $-$19.226 | 2.953 | 0.036 | 9.90 | 5 498.00 | 0.000 |
| **PARRO** | $-$0.043 | 0.000 | 60.041 | $-$85.898 | 3.945 | $-$1.938 | 109.36 | 1 307 425.00 | 0.000 |
| **LKOH** | 0.048 | 0.000 | 14.344 | $-$25.822 | 1.913 | $-$1.223 | 24.56 | 54 333.25 | 0.000 |
| **PSX** | 0.019 | 0.079 | 20.026 | $-$17.276 | 2.131 | $-$0.165 | 12.21 | 9 810.46 | 0.000 |
| **CSUAY** | 0.011 | 0.000 | 10.053 | $-$14.090 | 2.083 | $-$0.170 | 6.40 | 1 344.36 | 0.000 |
| **VLO** | 0.036 | 0.091 | 27.157 | $-$21.345 | 2.459 | 0.033 | 15.05 | 16 752.73 | 0.000 |
| **RELI** | 0.068 | 0.000 | 13.729 | $-$14.103 | 1.697 | 0.088 | 11.48 | 8 300.01 | 0.000 |
| **TTE** | 0.000 | 0.059 | 14.216 | 19.627 | 1.821 | $-$0.832 | 18.40 | 27 686.74 | 0.000 |
| **V0386** | $-$0.013 | 0.000 | 9.447 | $-$9.433 | 1.795 | 0.058 | 6.77 | 1 644.21 | 0.000 |
| **PTT** | 0.004 | 0.000 | 13.596 | $-$29.214 | 1.709 | $-$1.560 | 38.20 | 144 151.40 | 0.000 |
| **MPC** | 0.045 | 0.151 | 18.755 | $-$31.483 | 2.530 | $-$0.859 | 18.82 | 29 208.53 | 0.000 |
| **IOC** | 0.040 | 0.000 | 8.796 | $-$17.264 | 1.980 | $-$0.441 | 7.95 | 2 917.80 | 0.000 |
| **CVX** | 0.012 | 0.047 | 20.490 | $-$25.006 | 1.824 | $-$0.942 | 29.72 | 82 811.57 | 0.000 |
| **EQNR** | $-$0.004 | 0.000 | 12.491 | $-$23.643 | 2.176 | $-$0.612 | 11.63 | 8 774.12 | 0.000 |
| **SHEL** | $-$0.003 | 0.034 | 17.965 | $-$18.841 | 1.907 | $-$0.814 | 1.90 | 29 964.54 | 0.000 |
| **GAZP** | 0.007 | 0.000 | 22.275 | $-$36.320 | 2.105 | $-$2.553 | 60.03 | 378 366.30 | 0.000 |
| **NEE** | 0.040 | 0.119 | 12.837 | $-$14.403 | 1.508 | $-$0.504 | 13.65 | 13 198.99 | 0.000 |
| **NGG** | $-$0.004 | 0.020 | 13.613 | $-$15.408 | 1.381 | $-$0.737 | 17.85 | 25 685.79 | 0.000 |
| **EBKG** | 0.032 | 0.000 | 22.314 | $-$12.516 | 2.512 | 0.553 | 8.08 | 3 120.94 | 0.000 |
| **ONGC** | 0.008 | 0.000 | 16.982 | $-$18.422 | 2.159 | $-$0.567 | 14.11 | 14 385.24 | 0.000 |
| **SU** | 0.006 | 0.031 | 21.646 | $-$24.145 | 2.323 | $-$0.350 | 18.42 | 27 510.20 | 0.000 |
| **EXC** | 0.013 | 0.104 | 16.546 | $-$30.648 | 1.637 | $-$2.640 | 60.45 | 384 145.70 | 0.000 |
| **RWEG** | 0.001 | 0.000 | 15.376 | $-$18.992 | 2.044 | $-$0.495 | 10.15 | 6 005.47 | 0.000 |
| **E** | $-$0.018 | 0.060 | 12.681 | $-$24.632 | 1.839 | $-$1.423 | 22.48 | 44 752.36 | 0.000 |
| **V0883** | 0.013 | 0.000 | 13.446 | $-$18.912 | 2.138 | $-$0.127 | 9.04 | 4 224.08 | 0.000 |
| **ITA** | 0.037 | 0.078 | 11.589 | $-$15.948 | 1.375 | $-$0.832 | 19.87 | 33 165.30 | 0.000 |
| **XAR** | 0.042 | 0.073 | 9.651 | $-$14.281 | 1.433 | $-$0.714 | 13.74 | 13 544.08 | 0.000 |
| **PPA** | 0.046 | 0.082 | 9.897 | $-$13.288 | 1.233 | $-$0.697 | 17.09 | 23 150.16 | 0.000 |
| **VIS** | 0.035 | 0.074 | 11.248 | $-$12.173 | 1.230 | $-$0.684 | 15.81 | 19 162.54 | 0.000 |
| **IYJ** | 0.036 | 0.077 | 11.607 | $-$13.310 | 1.204 | $-$0.637 | 17.34 | 23 908.00 | 0.000 |
| **FIDU** | 0.035 | 0.075 | 11.593 | $-$12.231 | 1.223 | $-$0.658 | 16.38 | 20 863.15 | 0.000 |
| **EXI** | 0.027 | 0.060 | 9.898 | $-$10.794 | 1.115 | 0.784 | 15.90 | 19 500.72 | 0.000 |
| The S2 Appendix presents the descriptive statistics for the sample of companies and ETFs, including measures such as the mean, median, maximum, minimum, standard deviation, skewness, kurtosis, and the results of the Jarque-Bera (JB) normality test. The JB test values are significant for almost all companies and ETFs (p-value < 0.05), indicating that the return distributions deviate from normality. | | | | | | | | | |
