# Supplementary material for: Geopolitical risk contagion across strategic sectors: Nonlinear evidence from defense, cybersecurity, energy, and raw materials
Source: PLoS One. 2025 Sep 2;20(9):e0330557. doi: 10.1371/journal.pone.0330557 (PMC12404389; doi:10.1371/journal.pone.0330557)
Supplement: S3 Appendix — (DOCX) [file pone.0330557.s005.docx]

| **S3 APPENDIX Stationarity tests** | | | | | | | | | | | | |
| --- | --- | --- | --- | --- | --- | --- | --- | --- | --- | --- | --- | --- |
|  | **ADF** | | | | **PP** | | | | **KPSS** | | | |
|  | ***level*** | | ***1st Diff*** | | ***level*** | | ***1st Diff*** | | ***level*** | | ***1st Diff*** | |
|  | t-Statistic | p-Value | t-Statistic | p-Value | t-Statistic | p-Value | t-Statistic | p-Value | LM-Statistic | p-Value | LM-Statistic | p-Value |
| **LMT** | $-$53.0553 | 0.0001 | $-$23.3475 | 0.0000 | $-$53.0772 | 0.0001 | $-$827.5878 | 0.0001 | 0.0903 | 0.1748 | 0.0431 | 0.9916 |
| **RTX** | $-$14.8643 | 0.0000 | $-$23.3263 | 0.0000 | $-$53.9512 | 0.0001 | $-$959.9248 | 0.0001 | 0.0655 | 0.4689 | 0.0339 | 0.9946 |
| **NOC** | $-$55.2908 | 0.0001 | $-$25.2781 | 0.0000 | $-$55.7310 | 0.0001 | $-$888.6213 | 0.0001 | 0.1717 | 0.0922 | 0.0368 | 0.9940 |
| **BA** | $-$32.9335 | 0.0000 | $-$24.7166 | 0.0000 | $-$50.0967 | 0.0001 | $-$693.5524 | 0.0001 | 0.0968 | 0.7939 | 0.0254 | 0.9971 |
| **GD** | $-$53.3747 | 0.0001 | $-$21.4997 | 0.0000 | $-$53.3836 | 0.0001 | $-$1807.6850 | 1.0000 | 0.0768 | 0.2409 | 0.0552 | 0.9921 |
| **BAES** | $-$51.1294 | 0.0001 | $-$20.3477 | 0.0000 | $-$51.1517 | 0.0001 | $-$1387.6290 | 1.0000 | 0.1358 | 0.1546 | 0.5000 | 0.9060 |
| **V2357** | $-$52.4090 | 0.0001 | $-$22.0871 | 0.0000 | $-$52.5644 | 0.0001 | $-$745.4857 | 0.0001 | 0.0205 | 0.9226 | 0.0414 | 0.9884 |
| **LHX** | $-$55.4002 | 0.0001 | $-$24.7441 | 0.0000 | $-$55.4743 | 0.0001 | $-$751.5776 | 0.0001 | 0.1417 | 0.2180 | 0.0224 | 0.9979 |
| **AIR** | $-$20.1481 | 0.0000 | $-$24.0316 | 0.0000 | $-$50.1048 | 0.0001 | $-$537.8798 | 0.0001 | 0.0294 | 0.3216 | 0.0602 | 0.9840 |
| **LDOF** | $-$50.9201 | 0.0001 | $-$23.1288 | 0.0000 | $-$51.0646 | 0.0001 | $-$673.3304 | 0.0001 | 0.2658 | 0.1887 | 0.0292 | 0.9855 |
| **TCFP** | $-$50.4900 | 0.0001 | $-$19.9015 | 0.0000 | $-$50.4744 | 0.0001 | $-$601.4839 | 0.0001 | 0.0714 | 0.1091 | 0.1352 | 0.9979 |
| **HII** | $-$53.6598 | 0.0001 | $-$20.9088 | 0.0000 | $-$53.6743 | 0.0001 | $-$1383.9080 | 1.0000 | 0.2078 | 0.5615 | 0.0476 | 0.9831 |
| **LDOS** | $-$52.5943 | 0.0001 | $-$25.3461 | 0.0000 | v52.5964 | 0.0001 | $-$2789.2480 | 1.0000 | 0.0415 | 0.1491 | 0.0333 | 0.9955 |
| **BAH** | $-$53.2159 | 0.0001 | $-$20.4162 | 0.0000 | $-$53.2593 | 0.0001 | $-$706.2484 | 0.0001 | 0.1003 | 0.0404 | 0.0406 | 0.9861 |
| **RR** | $-$51.2001 | 0.0001 | $-$19.8086 | 0.0000 | $-$51.1810 | 0.0001 | $-$793.8044 | 0.0001 | 0.3853 | 0.8042 | 0.0207 | 0.9943 |
| **CACI** | $-$35.6667 | 0.0000 | $-$23.2669 | 0.0000 | $-$55.6950 | 0.0001 | $-$973.8907 | 0.0001 | 0.0642 | 0.0931 | 0.0271 | 0.9924 |
| **RHMG** | $-$47.4452 | 0.0001 | $-$20.9732 | 0.0000 | $-$47.2159 | 0.0001 | $-$564.6554 | 0.0001 | 0.4891 | 0.0140 | 0.0641 | 0.9726 |
| **ESLT** | $-$55.8832 | 0.0001 | $-$22.7004 | 0.0000 | $-$56.0571 | 0.0001 | $-$773.7413 | 0.0001 | 0.0558 | 0.0551 | 0.0255 | 0.9977 |
| **HON** | $-$19.9038 | 0.0000 | $-$21.2600 | 0.0000 | $-$54.4394 | 0.0001 | $-$1754.8020 | 1.0000 | 0.0450 | 0.2710 | 0.0245 | 0.9879 |
| **GE** | $-$53.5513 | 0.0001 | $-$22.7361 | 0.0000 | $-$53.5884 | 0.0001 | $-$720.7698 | 0.0001 | 0.4441 | 0.8155 | 0.0260 | 0.9973 |
| **SAF** | $-$21.5428 | 0.0000 | $-$21.4124 | 0.0000 | $-$51.0610 | 0.0001 | $-$633.4370 | 0.0001 | 0.0624 | 0.1574 | 0.0311 | 0.9919 |
| **SAABBs** | $-$49.1688 | 0.0001 | $-$20.5083 | 0.0000 | $-$49.1632 | 0.0001 | $-$1248.0320 | 1.0000 | 0.1820 | 0.0462 | 0.1306 | 0.9981 |
| **KBR** | $-$35.0289 | 0.0000 | $-$20.6003 | 0.0000 | $-$53.9667 | 0.0001 | $-$947.3540 | 0.0001 | 0.2004 | 0.7102 | 0.0274 | 0.9907 |
| **BAB** | $-$51.1929 | 0.0001 | $-$27.2866 | 0.0000 | $-$51.1829 | 0.0001 | $-$906.1863 | 0.0001 | 0.2690 | 0.5478 | 0.0301 | 0.9958 |
| **V7011** | $-$50.6649 | 0.0001 | $-$23.0521 | 0.0000 | $-$50.6331 | 0.0001 | $-$799.6498 | 0.0001 | 0.8059 | 0.2498 | 0.1031 | 0.9850 |
| **SAIC** | $-$55.5166 | 0.0001 | $-$22.9972 | 0.0000 | $-$55.5103 | 0.0001 | $-$1100.8390 | 1.0000 | 0.0875 | 0.3345 | 0.0506 | 0.9917 |
| **AM** | $-$52.9606 | 0.0001 | $-$21.3936 | 0.0000 | $-$52.9782 | 0.0001 | $-$932.4922 | 0.0001 | 0.1293 | 0.3410 | 0.0535 | 0.9764 |
| **TXT** | $-$53.8991 | 0.0001 | $-$17.3853 | 0.0000 | $-$53.8902 | 0.0001 | $-$560.9568 | 0.0001 | 0.0354 | 0.5341 | 0.0552 | 0.9841 |
| **PH** | $-$19.2964 | 0.0000 | $-$19.0860 | 0.0000 | $-$54.7681 | 0.0001 | $-$729.6719 | 0.0001 | 0.1106 | 0.0830 | 0.0214 | 0.9885 |
| **TDG** | $-$20.6662 | 0.0000 | $-$27.4969 | 0.0000 | $-$54.0831 | 0.0001 | $-$960.3732 | 0.0001 | 0.0198 | 0.0243 | 0.0365 | 0.9946 |
| **ASELS** | $-$54.6182 | 0.0001 | $-$23.3894 | 0.0000 | $-$54.6096 | 0.0001 | $-$1985.9880 | 1.0000 | 0.1352 | 0.0040 | 0.0604 | 0.9948 |
| **J** | $-$52.4097 | 0.0001 | $-$21.5143 | 0.0000 | $-$52.4577 | 0.0001 | $-$668.2882 | 0.0001 | 0.0862 | 0.4043 | 0.0240 | 0.9888 |
| **V047810** | $-$53.2546 | 0.0001 | $-$26.4426 | 0.0000 | $-$53.2546 | 0.0001 | $-$711.9050 | 0.0001 | 0.0994 | 0.6272 | 0.1414 | 0.9983 |
| **SRP** | $-$51.1438 | 0.0001 | $-$21.2806 | 0.0000 | $-$51.1433 | 0.0001 | $-$868.0737 | 0.0001 | 0.2645 | 0.5262 | 0.0594 | 0.9947 |
| **STEG** | $-$55.8141 | 0.0001 | $-$22.6584 | 0.0000 | $-$55.7829 | 0.0001 | $-$866.8757 | 0.0001 | 0.1066 | 0.6410 | 0.0442 | 0.9891 |
| **TDY** | $-$19.9242 | 0.0000 | $-$23.1992 | 0.0000 | $-$55.6955 | 0.0001 | $-$1821.5260 | 1.0000 | 0.0787 | 0.0603 | 0.0404 | 0.9950 |
| **OSK** | $-$52.9964 | 0.0001 | $-$20.7676 | 0.0000 | $-$52.9964 | 0.0001 | $-$1003.1960 | 0.0001 | 0.0342 | 0.5835 | 0.0385 | 0.9949 |
| **V7012** | $-$52.8047 | 0.0001 | $-$19.5440 | 0.0000 | $-$52.8067 | 0.0001 | $-$919.6897 | 0.0001 | 0.0891 | 0.2597 | 0.5000 | 0.9896 |
| **TKAG** | $-$34.5400 | 0.0000 | $-$18.4211 | 0.0000 | $-$50.7174 | 0.0001 | $-$907.5495 | 0.0001 | 0.0500 | 0.4400 | 0.0590 | 0.9707 |
| **BAJE** | $-$53.3295 | 0.0001 | $-$21.9686 | 0.0000 | $-$53.3287 | 0.0001 | $-$848.5213 | 0.0001 | 0.2008 | 0.0079 | 0.0655 | 0.9972 |
| **MSFT** | $-$60.0749 | 0.0001 | $-$21.2967 | 0.0000 | $-$62.2330 | 0.0001 | $-$689.6919 | 0.0001 | 0.0881 | 0.0069 | 0.0180 | 0.9899 |
| **AVGO** | $-$55.9586 | 0.0001 | $-$22.1148 | 0.0000 | $-$56.5049 | 0.0001 | $-$838.1100 | 0.0001 | 0.0881 | 0.0038 | 0.0462 | 0.9679 |
| **CSCO** | $-$57.5690 | 0.0001 | $-$24.6267 | 0.0000 | $-$57.5973 | 0.0001 | $-$829.3144 | 0.0001 | 0.0502 | 0.1996 | 0.0226 | 0.9917 |
| **IBM** | $-$54.5386 | 0.0001 | $-$22.1787 | 0.0000 | $-$54.5212 | 0.0001 | $-$712.0978 | 0.0001 | 0.3196 | 0.6503 | 0.0217 | 0.9960 |
| **PANW** | $-$52.4756 | 0.0001 | $-$22.3057 | 0.0000 | $-$52.4759 | 0.0001 | $-$633.4025 | 0.0001 | 0.0379 | 0.0347 | 0.0443 | 0.9911 |
| **FTNT** | $-$55.2522 | 0.0001 | $-$23.3391 | 0.0000 | $-$55.6109 | 0.0001 | $-$780.8102 | 0.0001 | 0.0541 | 0.0138 | 0.0238 | 0.9844 |
| **CHKP** | $-$55.5972 | 0.0001 | $-$21.7051 | 0.0000 | $-$55.7563 | 0.0001 | $-$997.6255 | 0.0001 | 0.0573 | 0.1333 | 0.0481 | 0.9878 |
| **ACN** | $-$16.8578 | 0.0000 | $-$21.8704 | 0.0000 | $-$57.4281 | 0.0001 | $-$684.0961 | 0.0001 | 0.0354 | 0.0669 | 0.0161 | 0.9984 |
| **AKAM** | $-$52.5656 | 0.0001 | $-$21.9703 | 0.0000 | $-$52.6454 | 0.0001 | $-$730.7566 | 0.0001 | 0.0693 | 0.8119 | 0.0499 | 0.9973 |
| **FFIV** | $-$54.3406 | 0.0001 | $-$24.2806 | 0.0000 | $-$54.3437 | 0.0001 | $-$1017.6970 | 1.0000 | 0.0845 | 0.3022 | 0.0290 | 0.9907 |
| **NTCT** | $-$55.8737 | 0.0001 | $-$20.9633 | 0.0000 | $-$55.9533 | 0.0001 | $-$871.8567 | 0.0001 | 0.0281 | 0.6949 | 0.0239 | 0.9918 |
| **RDWR** | $-$52.5360 | 0.0001 | $-$21.8306 | 0.0000 | $-$52.6216 | 0.0001 | $-$872.1067 | 0.0001 | 0.0624 | 0.7478 | 0.0266 | 0.9965 |
| **V4704** | $-$34.8616 | 0.0000 | $-$23.2733 | 0.0000 | $-$53.4590 | 0.0001 | $-$1201.3430 | 1.0000 | 0.0475 | 0.2203 | 0.0614 | 0.9949 |
| **BB** | $-$54.3027 | 0.0001 | $-$24.1261 | 0.0000 | $-$54.3024 | 0.0001 | $-$921.8701 | 0.0001 | 0.0355 | 0.7874 | 0.0589 | 0.9958 |
| **JNPR** | $-$54.7982 | 0.0001 | $-$23.7492 | 0.0000 | $-$55.1216 | 0.0001 | $-$723.5941 | 0.0001 | 0.0490 | 0.7584 | 0.0245 | 0.9948 |
| **QLYS** | $-$54.6530 | 0.0001 | $-$22.0272 | 0.0000 | $-$54.7556 | 0.0001 | $-$1760.5650 | 1.0000 | 0.0570 | 0.2517 | 0.1124 | 0.9912 |
| **TMICY** | $-$53.0479 | 0.0001 | $-$22.4107 | 0.0000 | $-$53.0687 | 0.0001 | $-$784.5454 | 0.0001 | 0.0408 | 0.3913 | 0.0987 | 0.9851 |
| **ALLT** | $-$53.1006 | 0.0001 | $-$21.4070 | 0.0000 | $-$53.0986 | 0.0001 | $-$869.1501 | 0.0001 | 0.2258 | 0.5717 | 0.0423 | 0.9868 |
| **PARRO** | $-$29.1220 | 0.0000 | $-$22.5181 | 0.0000 | $-$53.3786 | 0.0001 | $-$1033.7130 | 1.0000 | 0.1031 | 0.5630 | 0.0817 | 0.9925 |
| **XOM** | $-$53.9062 | 0.0001 | $-$23.3313 | 0.0000 | $-$53.9152 | 0.0001 | $-$694.3537 | 0.0001 | 0.1742 | 0.8507 | 0.0292 | 0.9939 |
| **LKOH** | $-$53.5580 | 0.0001 | $-$20.8595 | 0.0000 | $-$53.6001 | 0.0001 | $-$903.8739 | 0.0001 | 0.0451 | 0.1839 | 0.0969 | 0.9985 |
| **PSX** | $-$54.0436 | 0.0001 | $-$19.9292 | 0.0000 | $-$54.0583 | 0.0001 | $-$530.1933 | 0.0001 | 0.0335 | 0.6405 | 0.0566 | 0.9933 |
| **CSUAY** | $-$54.7587 | 0.0001 | $-$22.1080 | 0.0000 | $-$55.3769 | 0.0001 | $-$1351.1450 | 1.0000 | 0.1438 | 0.7728 | 0.0385 | 0.9883 |
| **VLO** | $-$54.2022 | 0.0001 | $-$22.8837 | 0.0000 | $-$54.1953 | 0.0001 | $-$616.4811 | 0.0001 | 0.0314 | 0.4473 | 0.0756 | 0.9912 |
| **RELI** | $-$52.9252 | 0.0001 | $-$21.4142 | 0.0000 | $-$52.9243 | 0.0001 | $-$1680.2280 | 1.0000 | 0.0941 | 0.0339 | 0.0851 | 0.9983 |
| **TTE** | $-$35.1199 | 0.0000 | $-$21.5004 | 0.0000 | $-$54.5133 | 0.0001 | $-$726.1041 | 0.0001 | 0.0487 | 0.9967 | 0.0862 | 0.9951 |
| **V0386** | $-$53.3500 | 0.0001 | $-$19.8000 | 0.0000 | $-$53.3828 | 0.0001 | $-$696.6095 | 0.0001 | 0.0425 | 0.7048 | 0.2846 | 0.9450 |
| **PTT** | $-$54.5757 | 0.0001 | $-$20.9715 | 0.0000 | $-$54.5544 | 0.0001 | $-$695.1065 | 0.0001 | 0.0851 | 0.9121 | 0.0206 | 0.9894 |
| **MPC** | $-$53.8418 | 0.0001 | $-$22.8400 | 0.0000 | $-$54.2315 | 0.0001 | $-$560.8879 | 0.0001 | 0.0673 | 0.3458 | 0.0316 | 0.9927 |
| **IOC** | $-$52.7620 | 0.0001 | $-$22.3946 | 0.0000 | $-$52.7830 | 0.0001 | $-$677.4140 | 0.0001 | 0.1723 | 0.2928 | 0.2789 | 0.9977 |
| **CVX** | $-$18.5563 | 0.0000 | $-$23.3265 | 0.0000 | $-$56.7621 | 0.0001 | $-$1232.9730 | 1.0000 | 0.0547 | 0.7317 | 0.0209 | 0.9985 |
| **EQNR** | $-$54.4853 | 0.0001 | $-$19.8884 | 0.0000 | $-$54.4706 | 0.0001 | $-$1201.3200 | 1.0000 | 0.0827 | 0.9144 | 0.0263 | 0.9959 |
| **SHEL** | $-$52.4934 | 0.0001 | $-$20.5424 | 0.0000 | $-$52.5011 | 0.0001 | $-$831.8669 | 0.0001 | 0.1025 | 0.9382 | 0.0345 | 0.9936 |
| **GAZP** | $-$50.8219 | 0.0001 | $-$23.5418 | 0.0000 | $-$50.9232 | 0.0001 | $-$775.5943 | 0.0001 | 0.0464 | 0.8693 | 0.0521 | 0.9846 |
| **NEE** | $-$55.0691 | 0.0001 | $-$22.8282 | 0.0000 | $-$55.0674 | 0.0001 | $-$1410.7330 | 1.0000 | 0.1149 | 0.1585 | 0.0263 | 0.9924 |
| **NGG** | $-$34.9591 | 0.0000 | $-$22.2528 | 0.0000 | $-$53.6929 | 0.0001 | $-$1239.0620 | 1.0000 | 0.0254 | 0.8835 | 0.0478 | 0.9974 |
| **EBKG** | $-$36.7699 | 0.0000 | $-$22.5868 | 0.0000 | $-$73.8778 | 0.0001 | $-$736.2529 | 0.0001 | 0.1734 | 0.4975 | 0.0241 | 0.9989 |
| **ONGC** | $-$56.2473 | 0.0001 | $-$21.5859 | 0.0000 | $-$56.2768 | 0.0001 | $-$791.7088 | 0.0001 | 0.1605 | 0.8398 | 0.0482 | 0.9943 |
| **SU** | $-$51.3688 | 0.0001 | $-$18.8328 | 0.0000 | $-$51.5316 | 0.0001 | $-$469.4420 | 0.0001 | 0.0618 | 0.8958 | 0.0330 | 0.9994 |
| **EXC** | $-$21.9345 | 0.0000 | $-$24.4236 | 0.0000 | $-$58.0472 | 0.0001 | $-$716.7915 | 0.0001 | 0.0369 | 0.6681 | 0.0194 | 0.9938 |
| **RWEG** | $-$53.1911 | 0.0001 | $-$22.6779 | 0.0000 | $-$53.1917 | 0.0001 | $-$1268.4220 | 1.0000 | 0.1449 | 0.9821 | 0.0662 | 0.9861 |
| **E** | $-$35.6904 | 0.0000 | $-$20.8161 | 0.0000 | $-$55.4023 | 0.0001 | $-$1552.9810 | 1.0000 | 0.0792 | 0.6152 | 0.0266 | 0.9958 |
| **V0883** | $-$51.8758 | 0.0001 | $-$20.0658 | 0.0000 | $-$51.8751 | 0.0001 | $-$649.5525 | 0.0001 | 0.1176 | 0.7494 | 0.0759 | 0.9997 |
| **ITA** | $-$19.4794 | 0.0000 | $-$24.3530 | 0.0000 | $-$53.0022 | 0.0001 | $-$1066.2340 | 1.0000 | 0.0308 | 0.1568 | 0.0258 | 0.9903 |
| **XAR** | $-$19.4369 | 0.0000 | $-$24.3268 | 0.0000 | $-$53.6599 | 0.0001 | $-$995.9818 | 0.0001 | 0.0258 | 0.1243 | 0.0234 | 0.9843 |
| **PPA** | $-$19.1819 | 0.0000 | $-$23.1744 | 0.0000 | $-$53.7510 | 0.0001 | $-$956.7091 | 0.0001 | 0.0256 | 0.0495 | 0.0249 | 0.9872 |
| **VIS** | $-$16.3822 | 0.0000 | $-$20.4515 | 0.0000 | $-$54.4554 | 0.0001 | $-$775.5067 | 0.0001 | 0.0289 | 0.1359 | 0.0208 | 0.9866 |
| **IYJ** | $-$16.6287 | 0.0000 | $-$20.6787 | 0.0000 | $-$56.0558 | 0.0001 | $-$761.6443 | 0.0001 | 0.0253 | 0.1123 | 0.0182 | 0.9906 |
| **FIDU** | $-$16.4292 | 0.0000 | $-$20.5741 | 0.0000 | $-$54.3902 | 0.0001 | $-$799.2814 | 0.0001 | 0.0301 | 0.1300 | 0.0208 | 0.9858 |
| **EXI** | $-$16.5243 | 0.0000 | $-$21.4619 | 0.0000 | $-$54.2518 | 0.0001 | $-$847.7980 | 0.0001 | 0.0399 | 0.2056 | 0.0185 | 0.9939 |
| Note: S3Appendix presents the results of unit root tests for stationarity, including the Augmented Dickey-Fuller (ADF), Phillips-Perron (PP), and Kwiatkowski-Phillips-Schmidt-Shin (KPSS) tests at both the level and first difference for the variables in the study. The t-statistics and p-values for the ADF and PP tests are reported to examine the null hypothesis of a unit root (non-stationarity), with a rejection of this null hypothesis indicating stationarity. For the KPSS test, the null hypothesis is stationarity, and rejection of this null hypothesis suggests non-stationarity. | | | | | | | | | | | | |
