# Supplementary figures and images for: Geopolitical risk contagion across strategic sectors: Nonlinear evidence from defense, cybersecurity, energy, and raw materials
Source: PLoS One. 2025 Sep 2;20(9):e0330557. doi: 10.1371/journal.pone.0330557 (PMC12404389; doi:10.1371/journal.pone.0330557)

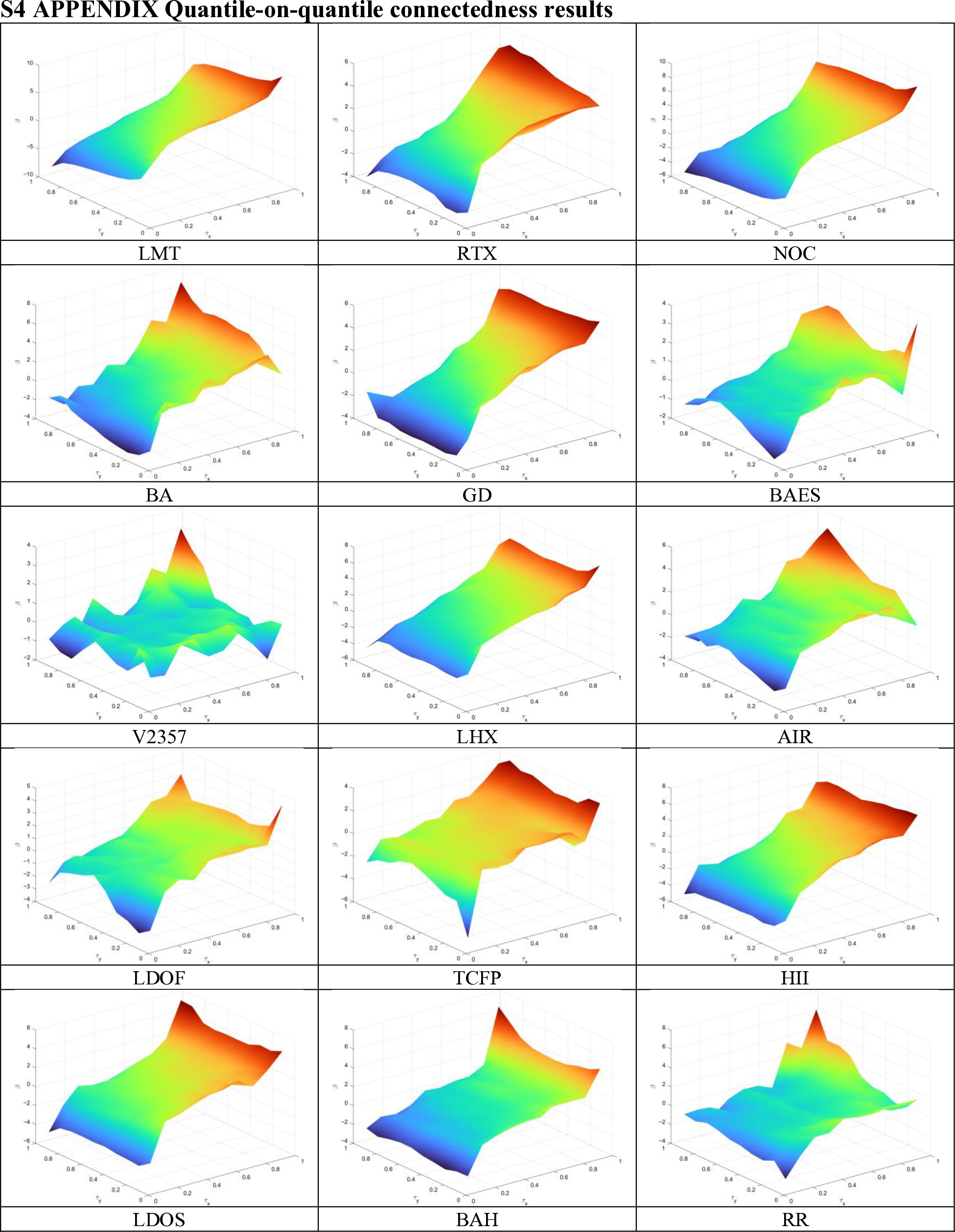

Supplement: S4 Appendix — (ZIP) [file pone.0330557.s006.zip › S4 Appendix-1.tif]

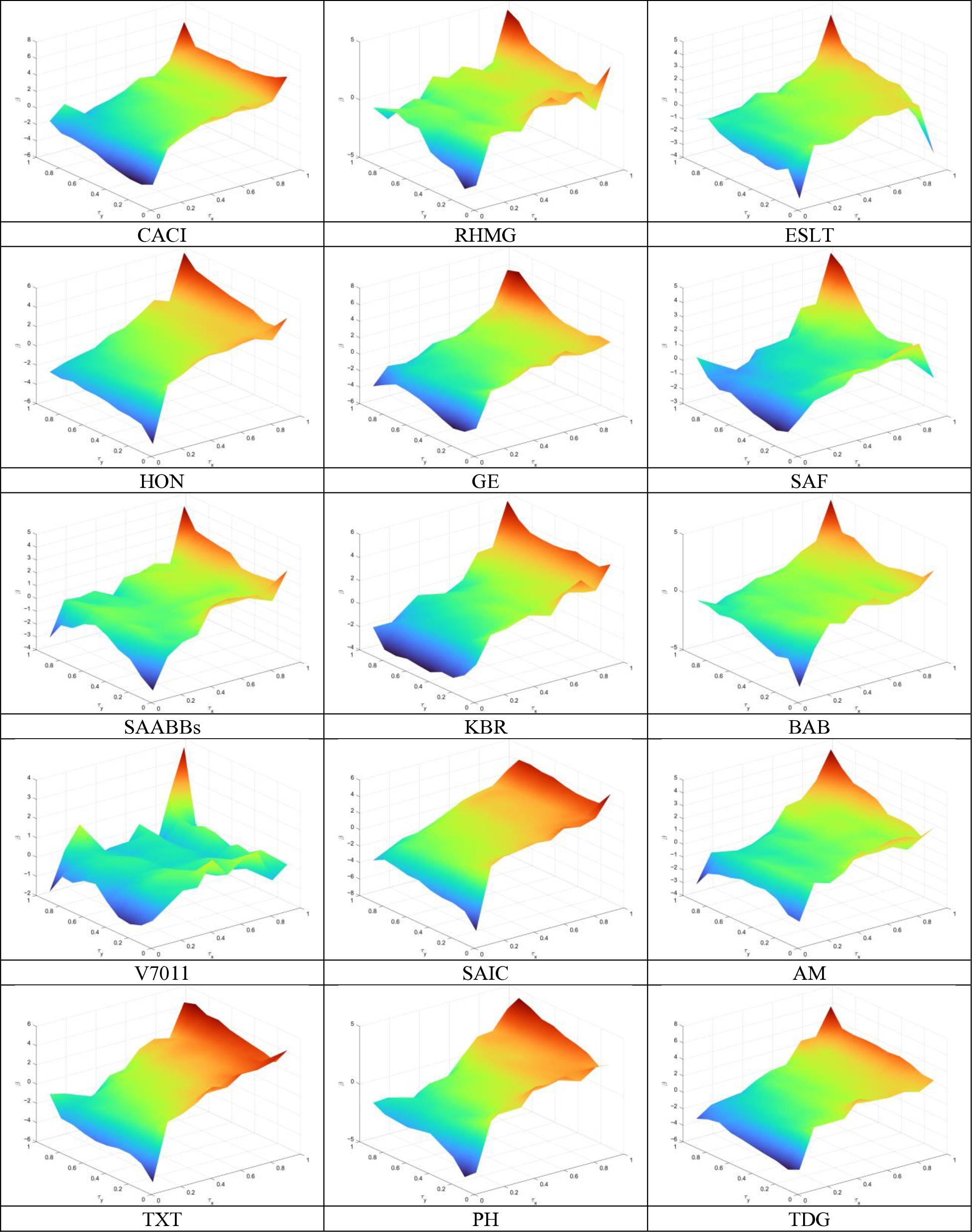

Supplement: S4 Appendix — (ZIP) [file pone.0330557.s006.zip › S4 Appendix-2.tif]

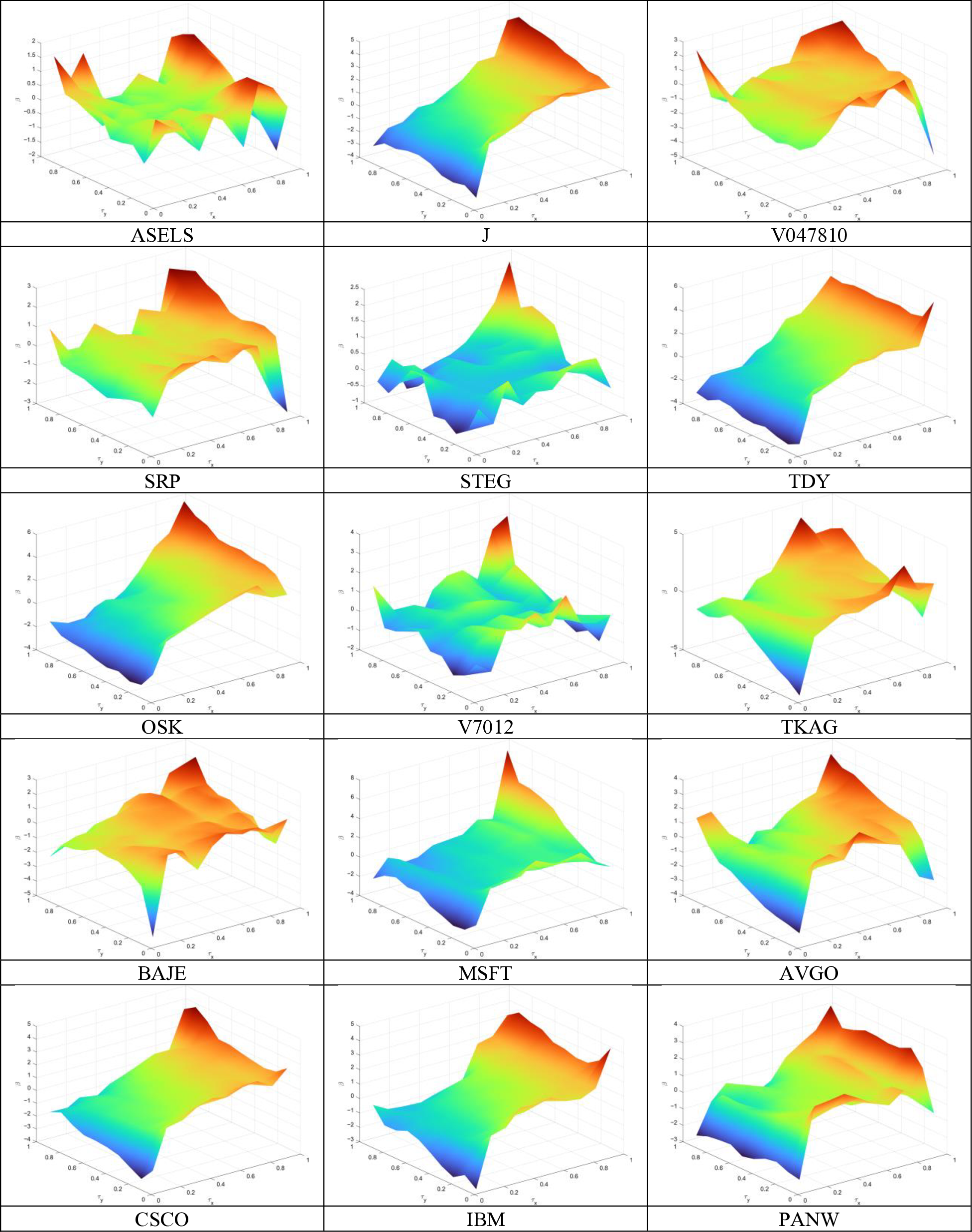

Supplement: S4 Appendix — (ZIP) [file pone.0330557.s006.zip › S4 Appendix-3.tif]

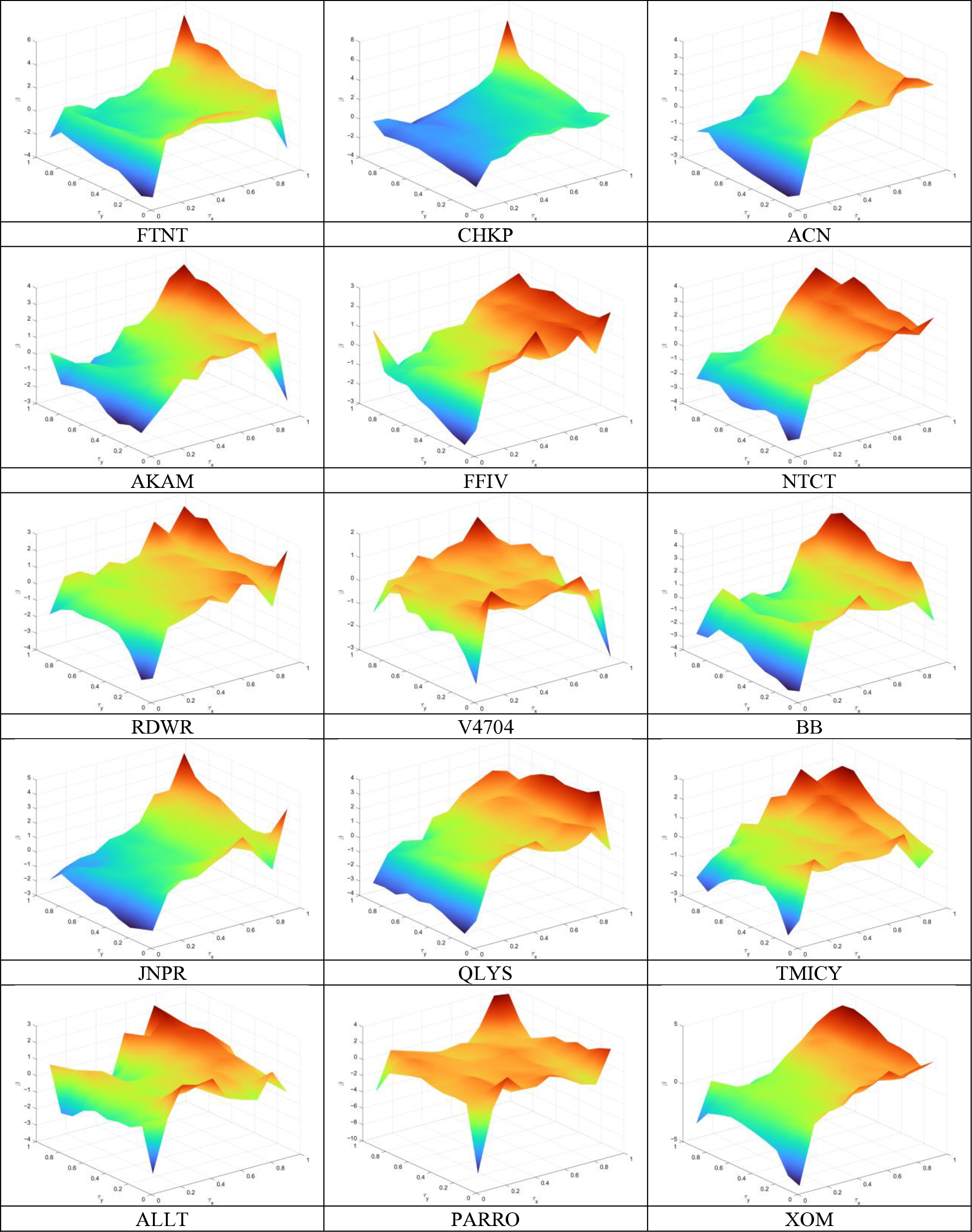

Supplement: S4 Appendix — (ZIP) [file pone.0330557.s006.zip › S4 Appendix-4.tif]

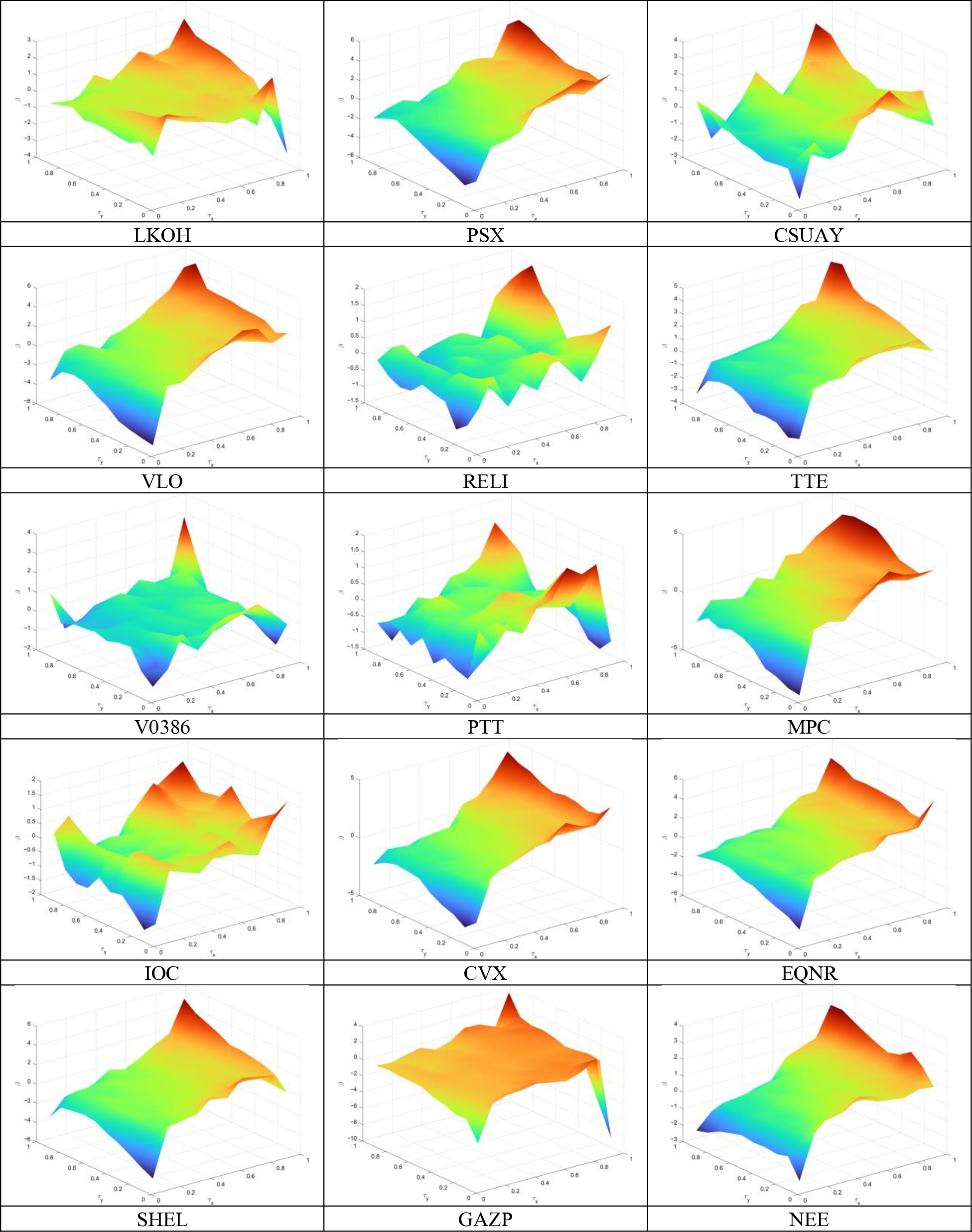

Supplement: S4 Appendix — (ZIP) [file pone.0330557.s006.zip › S4 Appendix-5.tif]

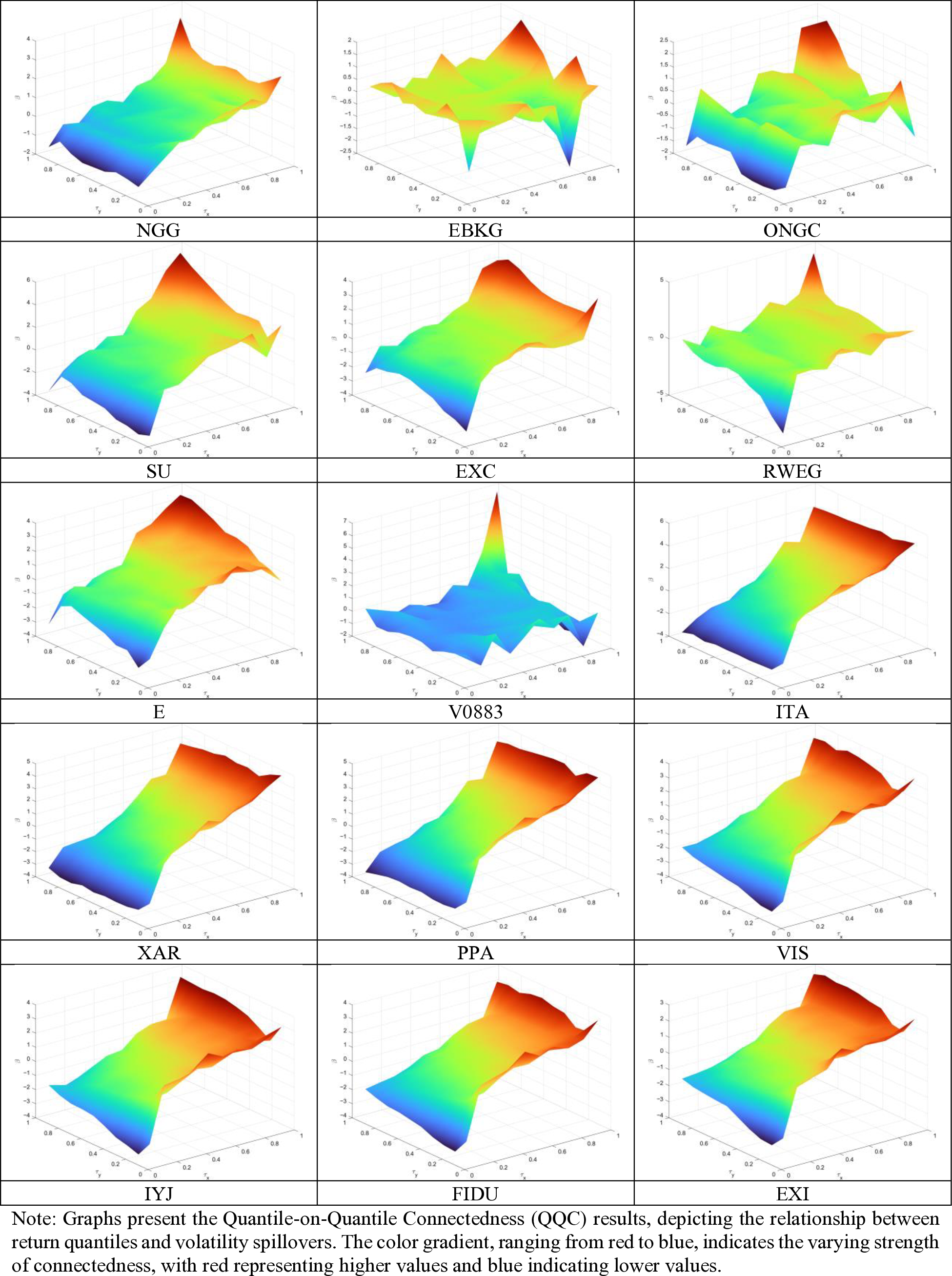

Supplement: S4 Appendix — (ZIP) [file pone.0330557.s006.zip › S4 Appendix-6.tif]

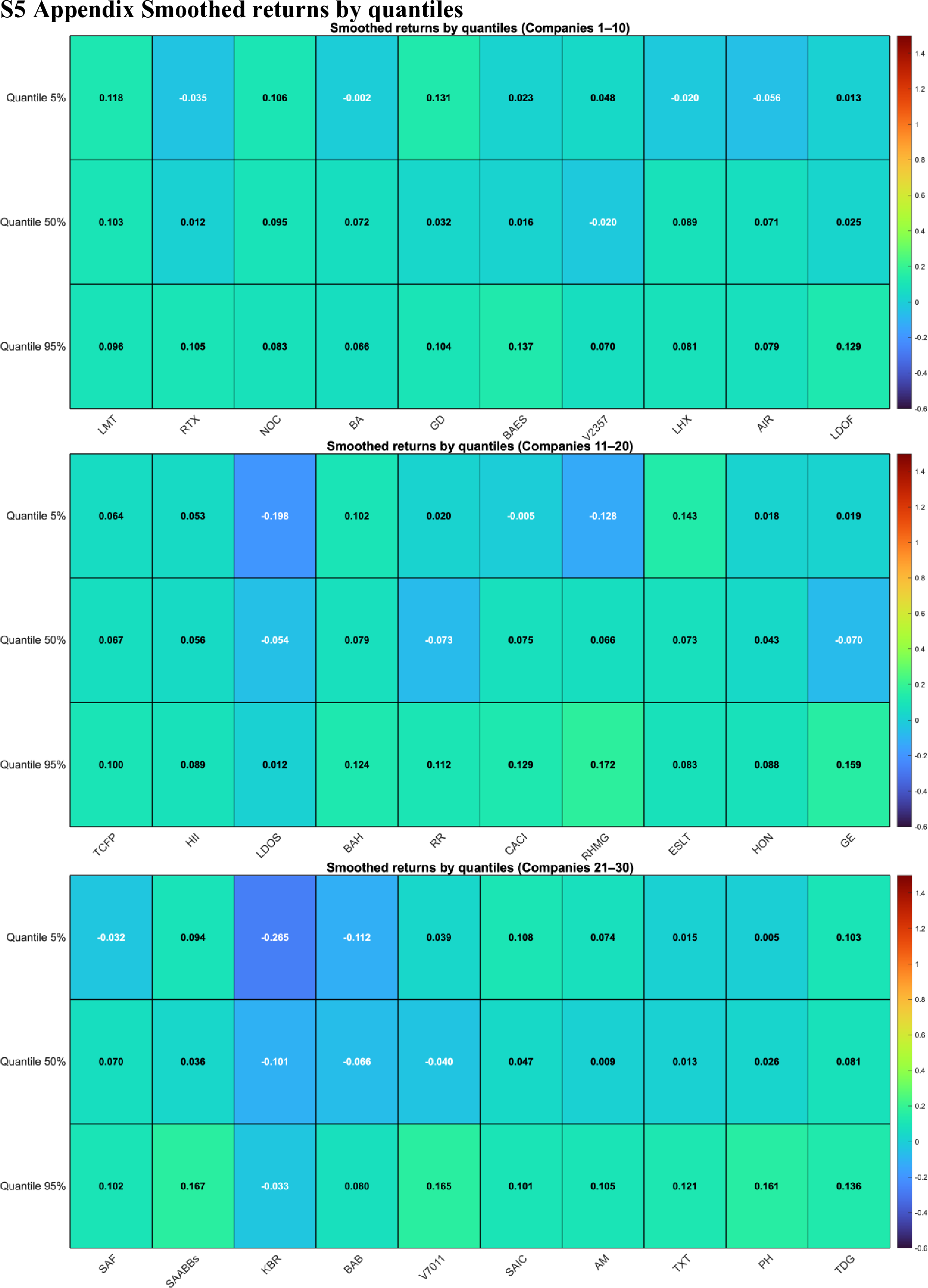

Supplement: S5 Appendix — (ZIP) [file pone.0330557.s007.zip › S5 Appendix-1.tif]

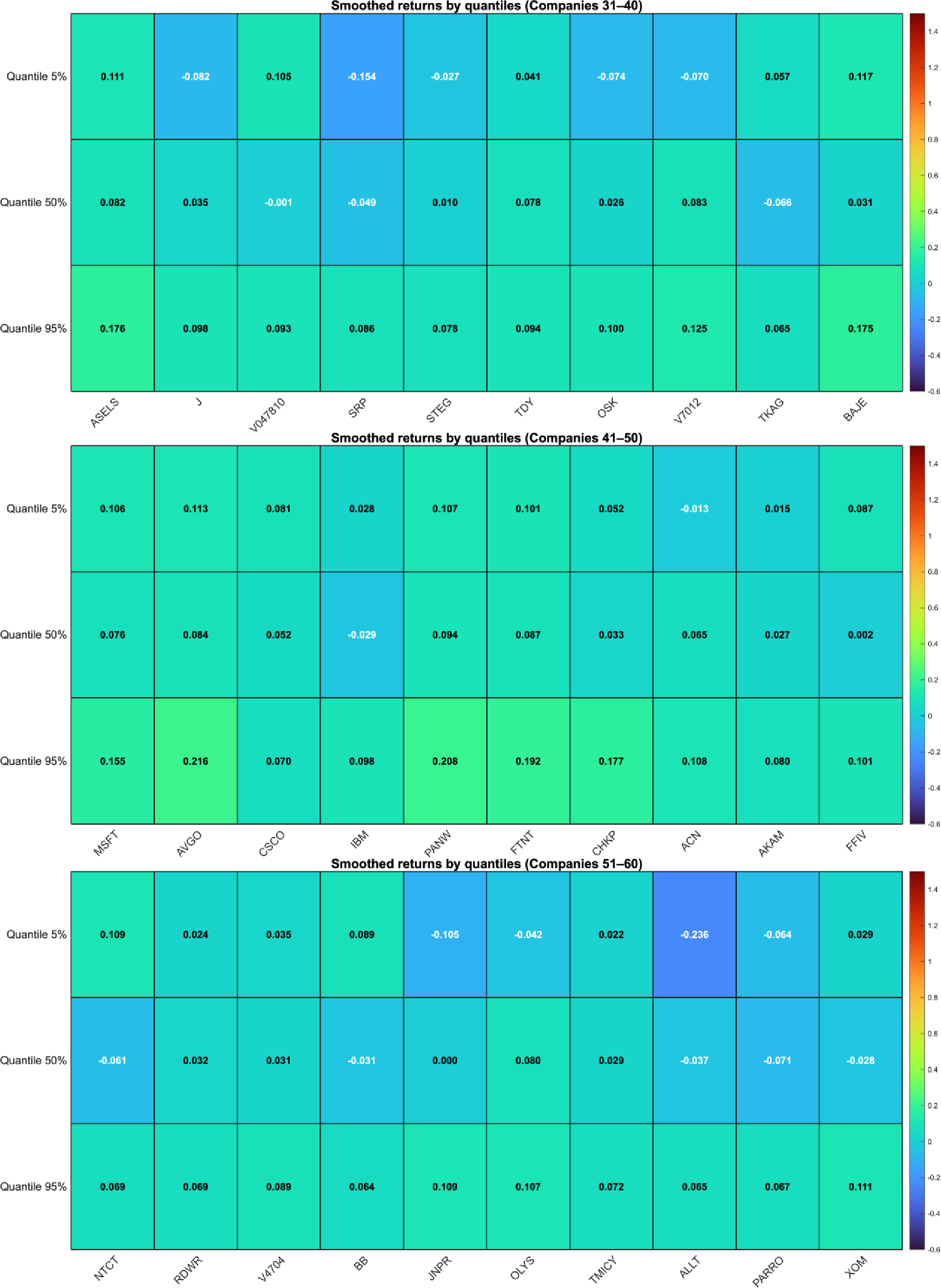

Supplement: S5 Appendix — (ZIP) [file pone.0330557.s007.zip › S5 Appendix-2.tif]

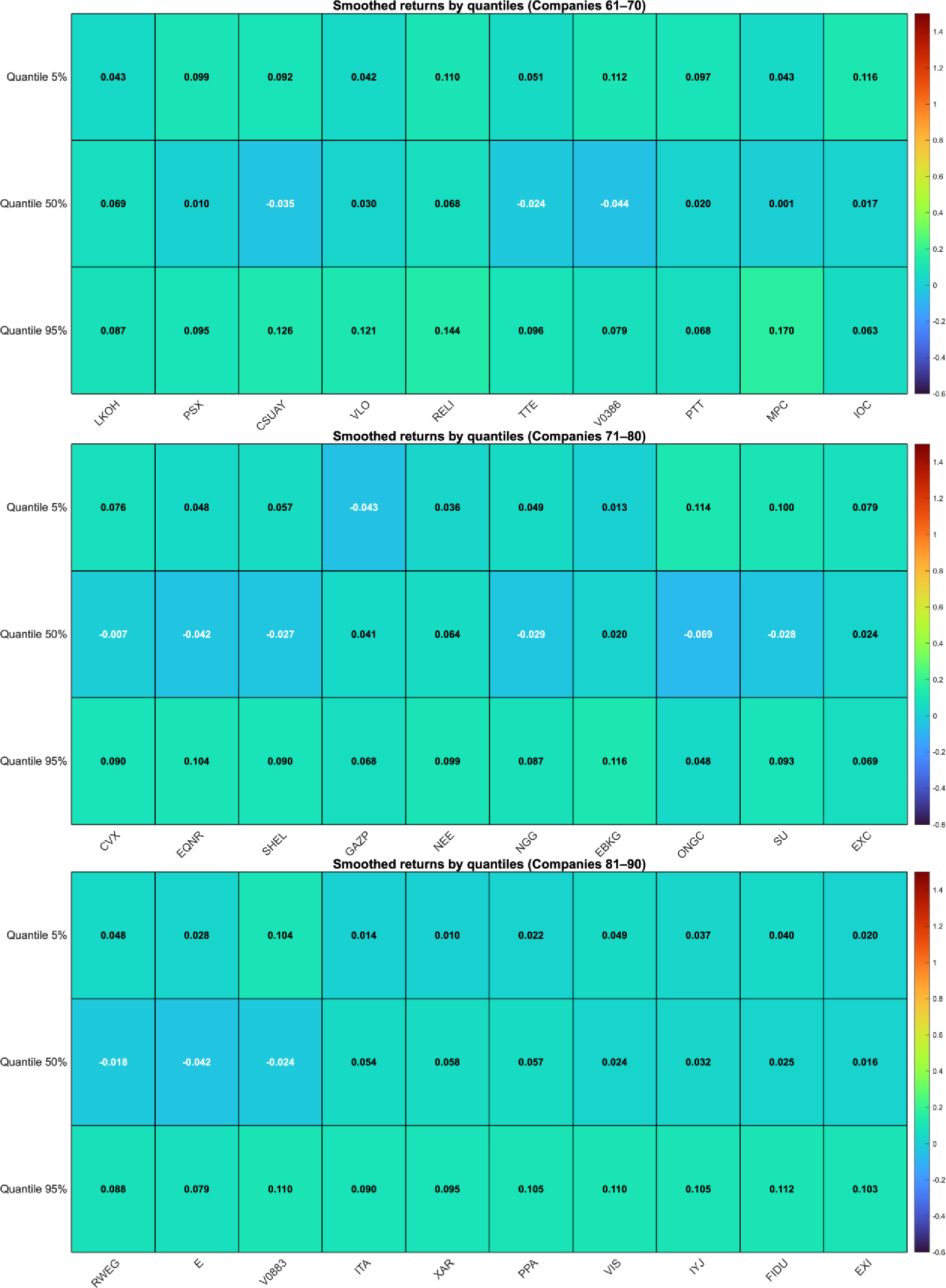

Supplement: S5 Appendix — (ZIP) [file pone.0330557.s007.zip › S5 Appendix-3.tif]

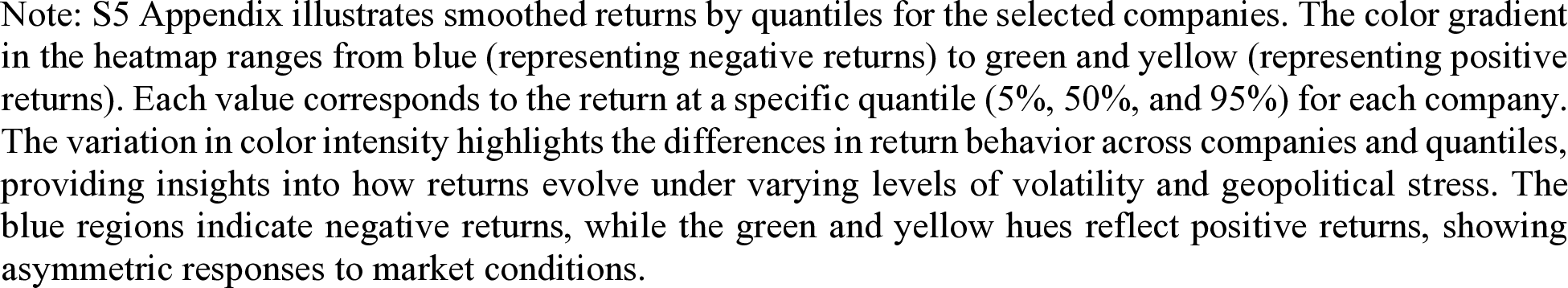

Supplement: S5 Appendix — (ZIP) [file pone.0330557.s007.zip › S5 Appendix-4.tif]

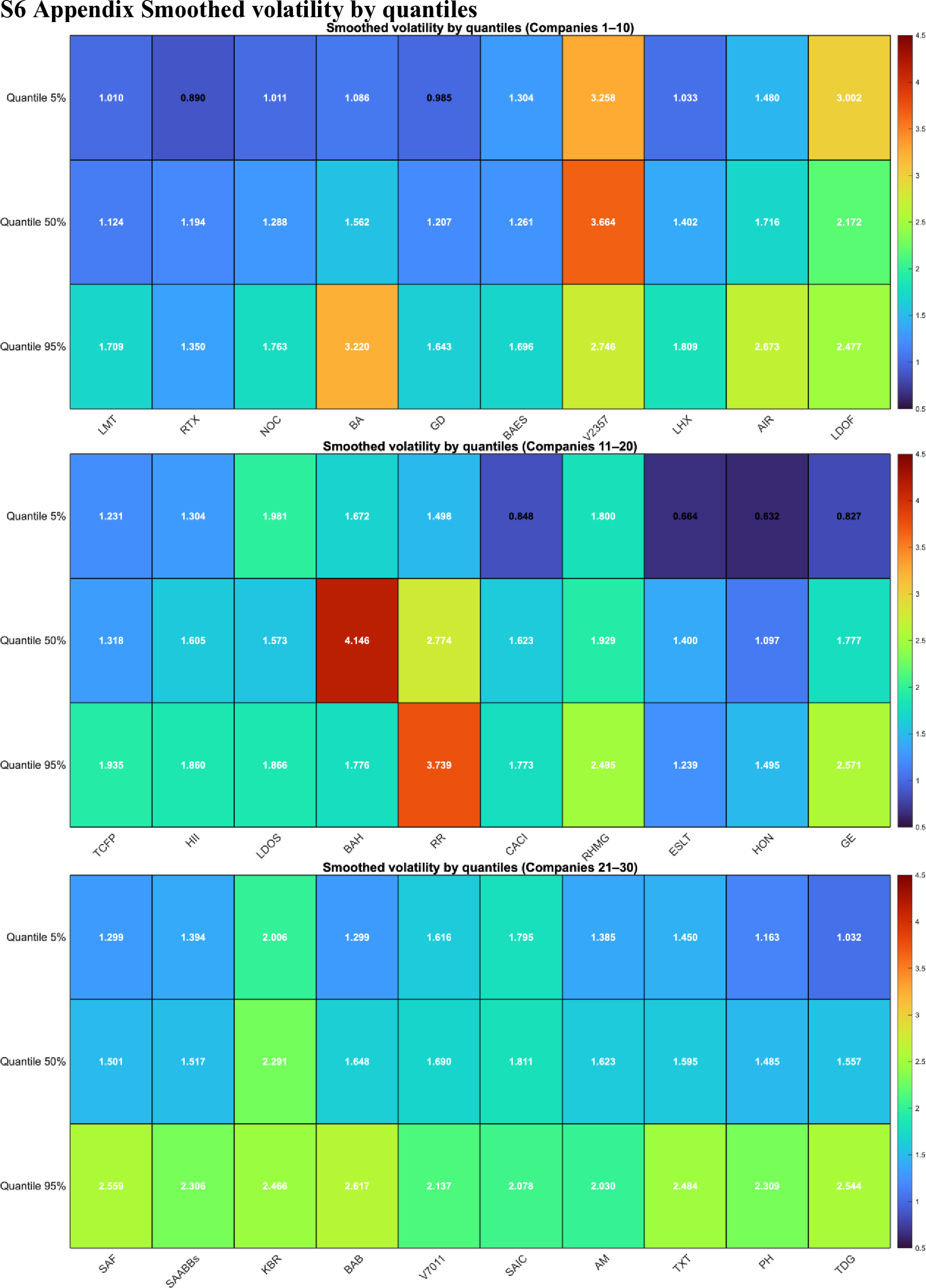

Supplement: S6 Appendix — (ZIP) [file pone.0330557.s008.zip › S6 Appendix-1.tif]

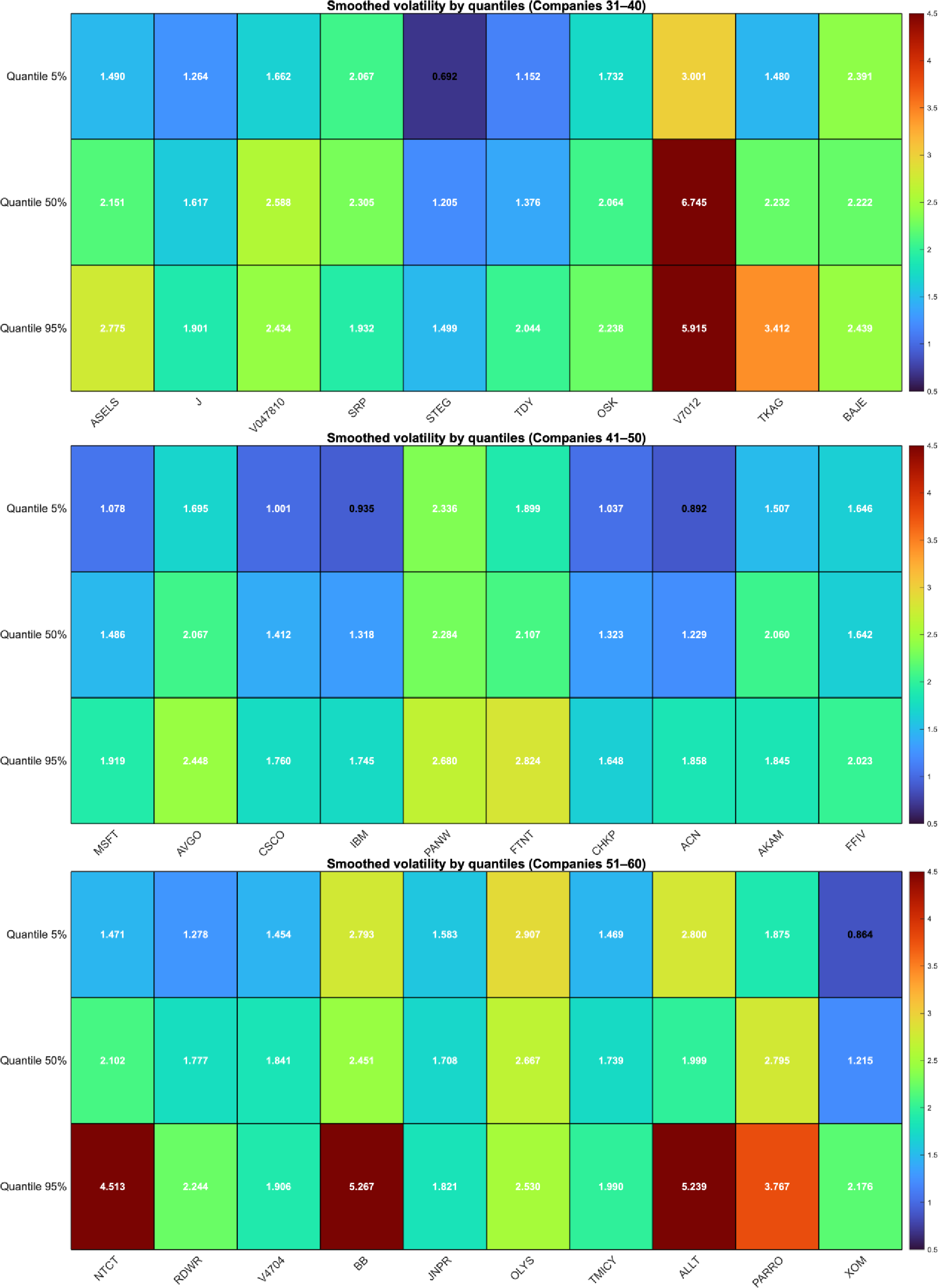

Supplement: S6 Appendix — (ZIP) [file pone.0330557.s008.zip › S6 Appendix-2.tif]

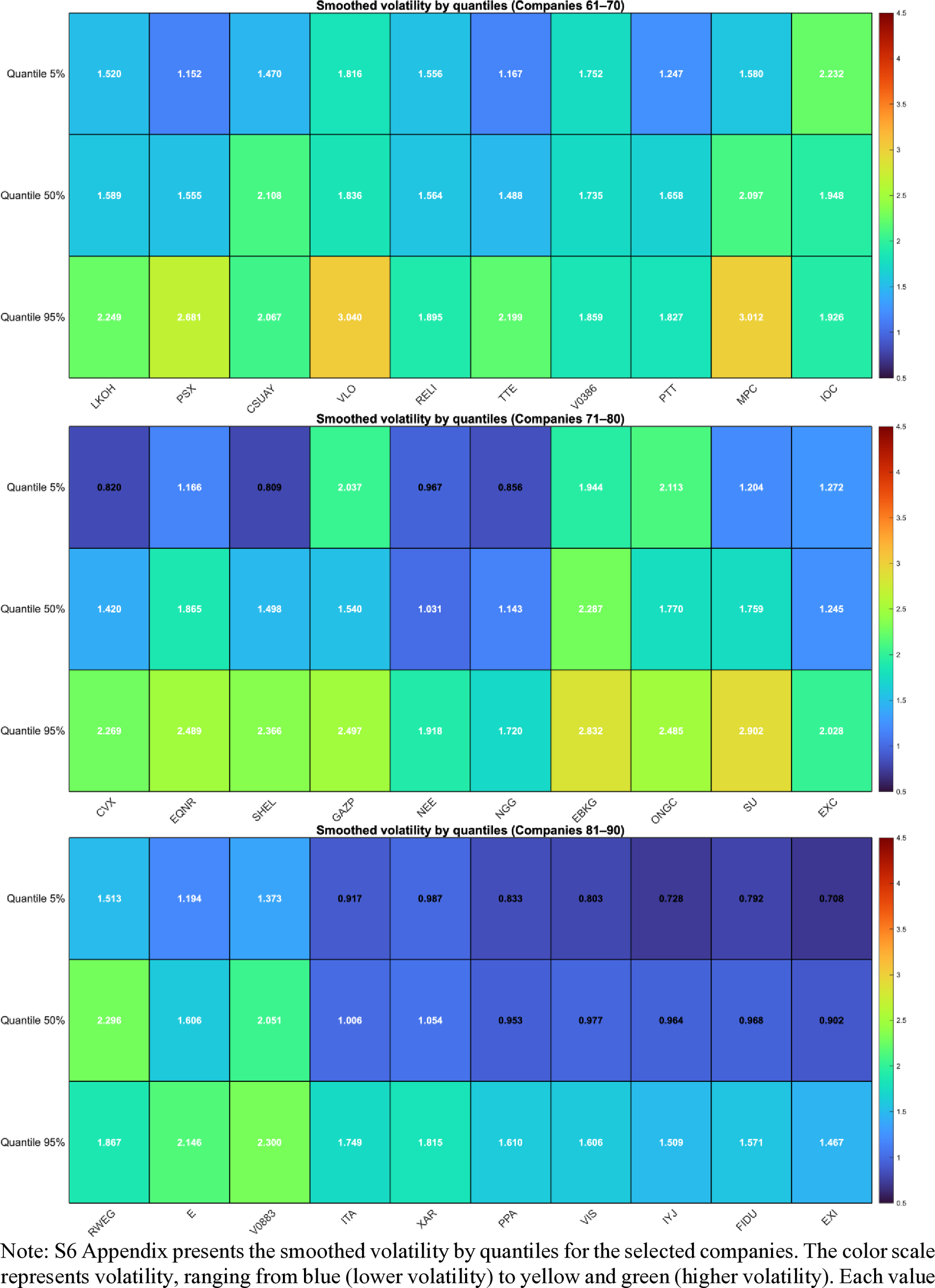

Supplement: S6 Appendix — (ZIP) [file pone.0330557.s008.zip › S6 Appendix-3.tif]

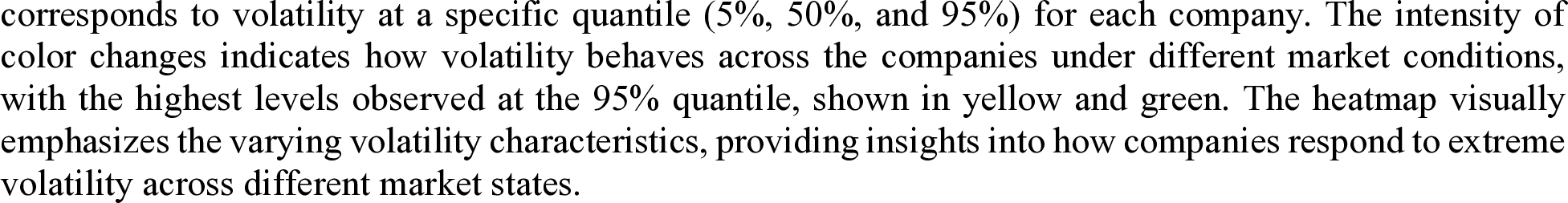

Supplement: S6 Appendix — (ZIP) [file pone.0330557.s008.zip › S6 Appendix-4.tif]

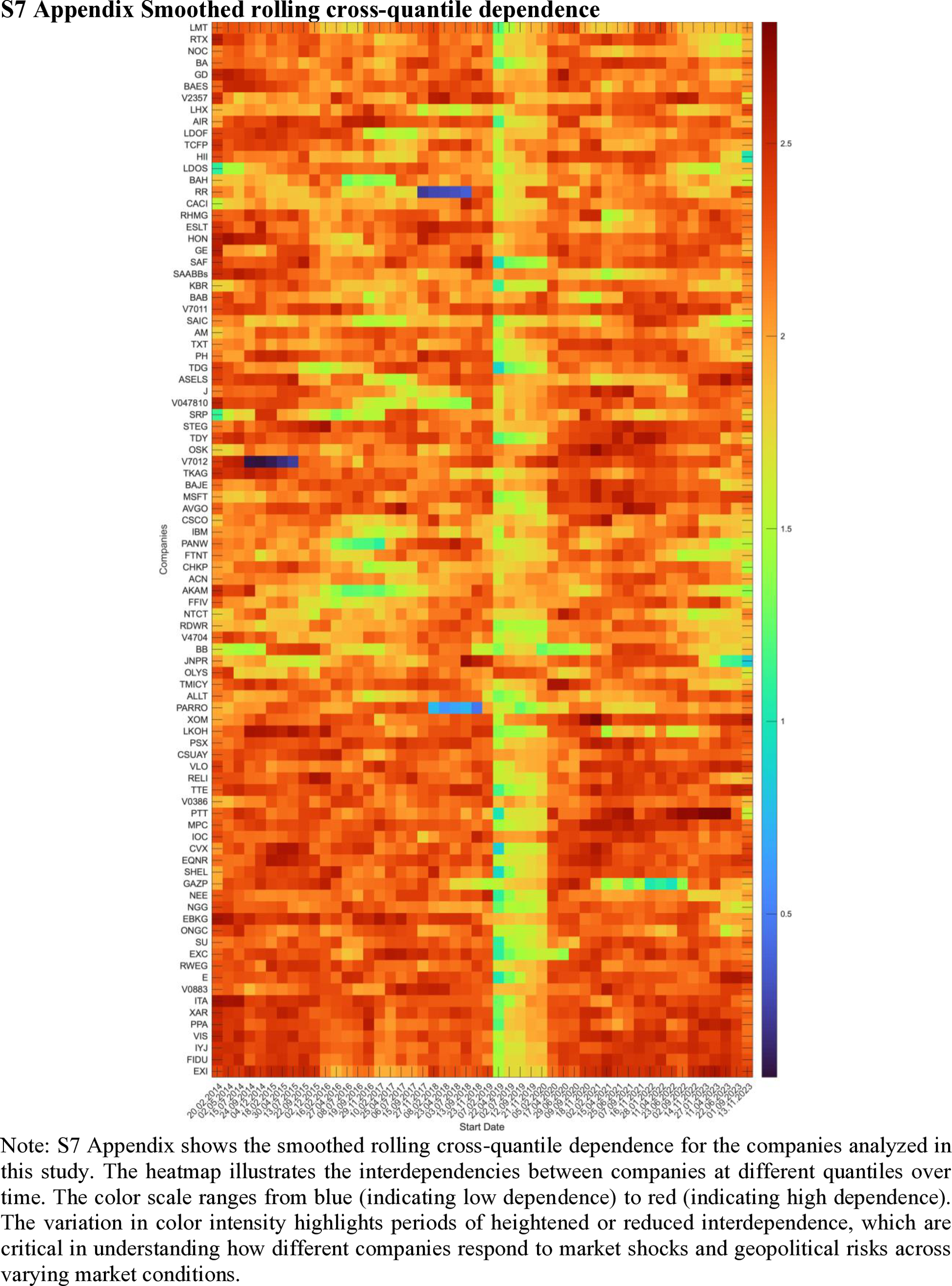

Supplement: S7 Appendix — (TIF) [file pone.0330557.s009.tif]
