# Supplementary material for: Geopolitical risk contagion across strategic sectors: Nonlinear evidence from defense, cybersecurity, energy, and raw materials
Source: PLoS One. 2025 Sep 2;20(9):e0330557. doi: 10.1371/journal.pone.0330557 (PMC12404389; doi:10.1371/journal.pone.0330557)
Supplement: Attachment — Submitted filename: Response to Reviewers.docx [file pone.0330557.s011.docx]

**Response to Reviewer #1**

We sincerely thank Reviewer 1 for the insightful comments and constructive suggestions that helped us improve the quality and clarity of our manuscript. Below, we provide a point-by-point response. All revisions are highlighted in the revised manuscript.

**Comment 1: The author(s) should carefully check that the references to the supplementary material are correct. For example, p. 15 states that “(e)stimates derived from the QQC method are reported in Appendix S4 for 5%, 50%, and 95% quantiles.” Apparently, these results are presented in S5. Furthermore, according to S5, the ESLT return (GD) under the 5th quantile is 0.149 (0.235), unlike what is reported in the article (p. 15). Therefore, the author(s) should carefully check that the information reported in the article corresponds to that in the appendices, that they are referring to the appendices correctly, and exclude appendices whose information is not discussed in the article.**

**Response:**
Thank you for this valuable observation. We carefully reviewed all references to supplementary appendices in the main text. We corrected the numbering and standardized the naming to align with PLOS ONE guidelines (e.g. Appendix S4 is now S4 Appendix). Additionally, numerical inconsistencies in the reported values of the QQC estimates have been corrected to reflect the actual results presented in the revised S5 Appendix. All such cross-references and numerical figures now match the supplementary material precisely.

**Comment 2: What are the criteria for the typology presented in Tables 1 and 2? For example, in Table 1, what range of return values do the categories “High”, “Moderate”, “Strong”, etc. correspond to?**

**Response:**
We appreciate this comment regarding typological clarity. We have added footnotes and an explanation in the Results section to indicate that these qualitative categories are derived from empirical coefficient thresholds. Specifically, they are based on the magnitude of QQC estimates across quantiles, as follows:

- Strong impact: coefficient ≥ |0.25|

- Moderate impact: |0.10| ≤ coefficient < |0.25|

- Low impact: coefficient < |0.10|

These cutoffs have been added in footnotes to Tables 1 and 2 and briefly explained in the narrative to ensure transparency and reproducibility.

**Comment 3: Throughout the article, it is not clear which variable (GPR, return or volatility) the author(s) are referring to when quantiles are mentioned. Initially, it seems that the author(s) are always referring to the quantile of the GPR. In particular, they are evaluating the behavior of companies' profitability and volatility at different quantiles of the GPR distribution. However, some contradictory statements call this idea into question. For example, page 15 states that the 95th quantile corresponds to “favorable geopolitical contexts”. However, on p. 17, this quantile is associated with “stress regimes”, which suggests that the authors are referring to the distribution of another variable. Please clarify these methodological issues.**

**Response:**
Thank you for highlighting this ambiguity. We have revised the text in the Methodology and Results sections to consistently clarify that the quantiles refer to the distribution of the GPR index, unless explicitly stated otherwise. The sentence on p.15 has been corrected to reflect that the lower quantiles (5%) correspond to low geopolitical uncertainty, while the higher quantiles (95%) indicate periods of elevated geopolitical stress. The contradictory phrasing between pages 15 and 17 has been removed to eliminate confusion and ensure consistent terminology throughout the manuscript.

**Comment 4: The meaning of the acronym ETF (exchange traded fund) is not provided in the paper.**

**Response:**
Thank you for catching this oversight. We have now defined the acronym ETF (Exchange-Traded Fund) at its first mention in the Introduction and ensured consistent formatting throughout the text.

**Additional clarifications (based on feedback):**

- The Introduction has been revised to more explicitly articulate the research gap, emphasizing the novelty of combining QQC and CiQ methods to investigate sectoral asymmetries under geopolitical uncertainty.

- We have expanded the Methodology section to include a theoretical justification for using the QQR and CiQ approaches. This addition clarifies how these methods capture nonlinear dynamics and regime-specific spillovers that traditional methods (GARCH, VAR) may miss.

- Equation formatting has been standardized throughout the manuscript to ensure consistency and meet PLOS formatting standards.

We are grateful for your constructive feedback and hope that the revisions meet your expectations and enhance the overall quality of the article.

**Response to Reviewer #2**

We sincerely thank Reviewer 2 for the insightful comments and constructive suggestions that helped us improve the quality and clarity of our manuscript. Below, we provide a point-by-point response. All revisions are highlighted in the revised manuscript.

**Comment 1: Stationarity Test Results**

*Response:* We acknowledge the importance of transparency regarding the stationarity properties of our data. We now provide a summary table in Appendix S3, indicating which series are stationary at levels and which required differencing, based on ADF, PP, and KPSS tests. We also specify the number of non-stationary series and the criteria used for transformation.

**Comment 2: Robustness Checks**

*Response:* Thank you for this valuable suggestion. We have added Section 5.3 to address robustness in relation to other major geopolitical events beyond the Russia–Ukraine conflict, such as the U.S. withdrawal from Afghanistan and recent Middle East escalations. These events correspond to volatility peaks observed in our models, supporting the regime-dependent effects of geopolitical shocks. We discuss these constraints and propose future methodological directions.

**Comment 3: Equation Formatting**

*Response:* We carefully reviewed all equations and ensured consistent formatting for all mathematical symbols. All subscripts, superscripts, quantile notations, and Greek letters are now uniformly presented to enhance readability and clarity.

**Comment 4: Terminology Consistency in Tables**

*Response:* Thank you for highlighting this inconsistency. We have revised the descriptive labels in Tables 1 and 2 to harmonize terminology across sectors. Qualitative descriptors such as “Low,” “Moderate,” “High,” and “Extreme” are now used consistently based on predefined volatility thresholds, and a legend explaining these thresholds is provided beneath each table.

**Comment 5: Definition and Scope of GPR**

*Response:* As suggested, we now include a concise definition and characterization of the Geopolitical Risk Index (GPR) in the Methodology section. This clarification outlines the index’s composition (geopolitical tensions, wars, and threats) and data source (Caldara and Iacoviello), helping contextualize its role in our empirical design.

**Comment 6: Intersectoral Nature of GPR**

*Response:* We now explain more explicitly how the QQC and CiQ methodologies allow us to capture intersectoral transmission of geopolitical risk. By modeling spillovers and asymmetric dependencies between sectors under different quantiles, our framework effectively addresses this limitation of previous research.

**Comment 7: Sector-Specific Relevance of GPR**

*Response:* We now include a dedicated paragraph at the end of the literature review section that details the specific types of geopolitical risk affecting each sector. For example, defense is primarily influenced by military conflict and defense spending, cybersecurity by cyber threats and attacks, energy by supply chain disruptions and embargoes, and raw materials by trade restrictions and strategic dependencies.

**Comment 8: Cybersecurity as Safe Haven Assets**

*Response:* We expanded the discussion in the Results section to justify why cybersecurity companies may act as safe havens. This includes their counter-cyclical demand during periods of cyber instability, strategic relevance in national security, and investor perception of their defensive role in times of geopolitical uncertainty.

**Comment 9: Theoretical Foundations**

*Response:* We agree that a stronger theoretical framing is essential. We have now introduced a paragraph at the start of the Methodology section discussing the theoretical justification for nonlinear modeling in financial contagion. This includes concepts from regime-dependent risk transmission, tail dependence, and quantile-specific spillovers, grounding our methodological choices in established financial theory.

**Comment 10: Hypothesis Motivation and Research Gap**

*Response:* We revised the final part of the literature review to strengthen the theoretical and empirical motivation for our two hypotheses (H1 and H2). We now more clearly articulate the empirical inconsistencies and limitations in previous studies that fail to capture nonlinear and regime-specific effects of GPR, thereby justifying our proposed methodological approach and research questions.

**Comment 11: Dual-Profile Companies**

*Response:* We appreciate this point. While we did not isolate dual-profile companies (those active in both defense and energy) in our econometric modeling, we have now added a paragraph in the Discussion section acknowledging this limitation and proposing it as a direction for future research.
